# Evaluating Transcriptomic Biomarkers for rHuEPO Detection: Assessing the Impact of Exercise and Altitude Exposure

**DOI:** 10.1002/dta.70040

**Published:** 2026-03-05

**Authors:** Daria Obratov, Shaun Sutehall, Longhua Liu, Zhao Zhongying, Yannis Pitsiladis

**Affiliations:** ^1^ Department of Sports and Health Sciences Hong Kong Baptist University Kowloon Tong Hong Kong SAR China; ^2^ Centre for Exercise Science and Medicine (CESAME) Hong Kong Baptist University Kowloon Tong Hong Kong SAR China; ^3^ Research Institute for Sport and Exercise Sciences Liverpool John Moores University Liverpool UK; ^4^ School of Exercise and Health Shanghai University of Sport Shanghai China; ^5^ Department of Biology, Faculty of Science Hong Kong Baptist University Kowloon Tong Hong Kong SAR China

**Keywords:** altitude, antidoping, erythropoietin, exercise, transcriptomics

## Abstract

Recombinant human erythropoietin (rHuEPO) is often misused in endurance sports due to its potent erythropoietic effects. While transcriptomic biomarkers hold promise for detecting rHuEPO use beyond conventional testing windows, many proposed gene markers may also respond to physiological stimuli such as exercise or altitude. This study compared 153 previously reported rHuEPO‐responsive genes in whole blood with transcripts identified during exercise (GEPREP database) and high‐altitude exposure (four independent studies). For the exercise dataset, gene‐level statistical outputs were obtained directly from the GEPREP database, while biological relevance was calculated using Cohen's *d*. Analyses of altitude and rHuEPO datasets followed the original statistical procedures described in each study. Among the 153 rHuEPO‐responsive genes, 94 overlapped with altitude and 34 with exercise. However, 50 genes remained unaffected by either exercise or altitude stimuli. Enriched in post‐translational regulation and intracellular transport pathways, these genes represent promising candidate transcriptomic markers of rHuEPO administration. This work provides a refined gene panel that reduces the likelihood of false positives and requires further experimental validation before integration into RNA‐based detection tests.

## Graphical abstract

A two‐stage transcriptomic filter comparing rHuEPO, exercise and altitude responses reduced 153 candidate genes to 50 that were unaffected by physiological stimuli. These retained transcripts offer focused biomarker leads to strengthen antidoping detection of rHuEPO.

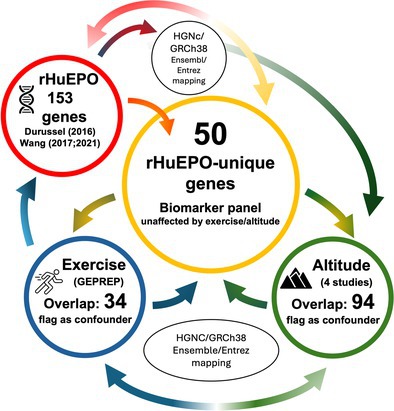

## 1 Introduction

Oxygen (O_2_) transport is a key determinant of endurance performance, as illustrated by the 1968 Mexico City Olympic Games, where athletes who resided and trained in high altitude environments performed substantially better than their lowlander competitors [1]. O_2_ delivery, specifically, haemoglobin concentration, was identified as a critical physiological variable for endurance performance in such conditions [1, 2]. Erythropoietin (EPO) is a glycoprotein hormone primarily produced by the kidneys that stimulates the maturation and differentiation of red blood cells [3]. EPO production is upregulated as part of the body's homeostatic response to hypoxia, enhancing erythropoiesis to restore O_2_ balance [3]. Recombinant human EPO (rHuEPO) was first synthesised in the late 1980s to treat anaemia, particularly in patients with chronic kidney disease [4]. Since then, rHuEPO has become the most widely used treatment for anaemia caused by various conditions [5].

Due to the strong haematopoietic effect of rHuEPO, its illicit use in sport has been shown to significantly enhance endurance performance by increasing red blood cell production [6]. These haematological changes were accompanied by ~8%–9% gains in VO_2_max and a ~54% improvement in submaximal time to exhaustion [6].

The Athlete Biological Passport (ABP), implemented by WADA in 2009, is a longitudinal profiling tool that tracks haematological markers (e.g., haemoglobin, haematocrit, reticulocytes and OFF‐score) to flag patterns consistent with blood doping [7]. It uses Bayesian statistical model to evaluate longitudinal changes in an athlete’s biomarkers relative to their individual baseline, accounting for analytical and intra‐individual biological variability. Potential confounding factors such as altitude exposure or medical conditions are considered during expert review of atypical profiles [8]. Despite significant efforts to detect the illicit use of rHuEPO in sports, important challenges remain. The ABP may be less sensitive to microdosing, as such regimens often lead to only subtle and transient changes in blood markers [9]. Training load, hydration status, illness and altitude exposure can complicate passport interpretation [8, 10, 11].

More recently, transcriptomic profiling has emerged as a promising strategy to address some of the limitations of existing testing methods. Transcriptomic profiling enables quantification of gene expression and the identification of differentially expressed genes (DEGs) in response to rHuEPO use, which could serve as biomarkers for doping detection [12]. Transcriptomic sequencing approaches, such as DEG sequencing and 3′ end sequencing, have been widely applied to characterise gene expression and identify disease‐related biomarkers [12]. Similarly, in the context of rHuEPO use, transcriptomic profiling may help identify DEGs that could serve as biomarkers for doping detection [11, 13, 14].

Previous whole‐blood transcriptomic studies in rHuEPO‐treated participants have identified multigene signatures with prolonged postdose regulation and extended detection windows beyond standard haematological markers [11, 13, 14]. These datasets provide a critical foundation for evaluating rHuEPO‐responsive genes and assessing their specificity against common confounders such as exercise and hypoxia.

Altitude‐induced hypoxia increases endogenous EPO production and alters whole‐blood gene expression, potentially generating transcriptomic profiles that mimic those of rHuEPO administration [15, 16, 17, 18]. Whole‐blood transcriptomic studies in athletes, sea‐level residents and mountaineers exposed to high altitude or hypoxia consistently show extensive modulation of genes involved in erythropoiesis, immune regulation and protein synthesis [15, 16, 17, 18] and therefore serve as critical comparators when assessing the specificity of putative rHuEPO‐responsive biomarkers.

To address the lack of curated, standardised resources for analysing exercise‐induced transcriptional responses, Sun et al. [19] established GEPREP, a publicly accessible RNA‐seq database of pre‐ and post‐exercise gene expression, with human data predominantly derived from skeletal muscle and blood across diverse interventions.

**FIGURE 1:**
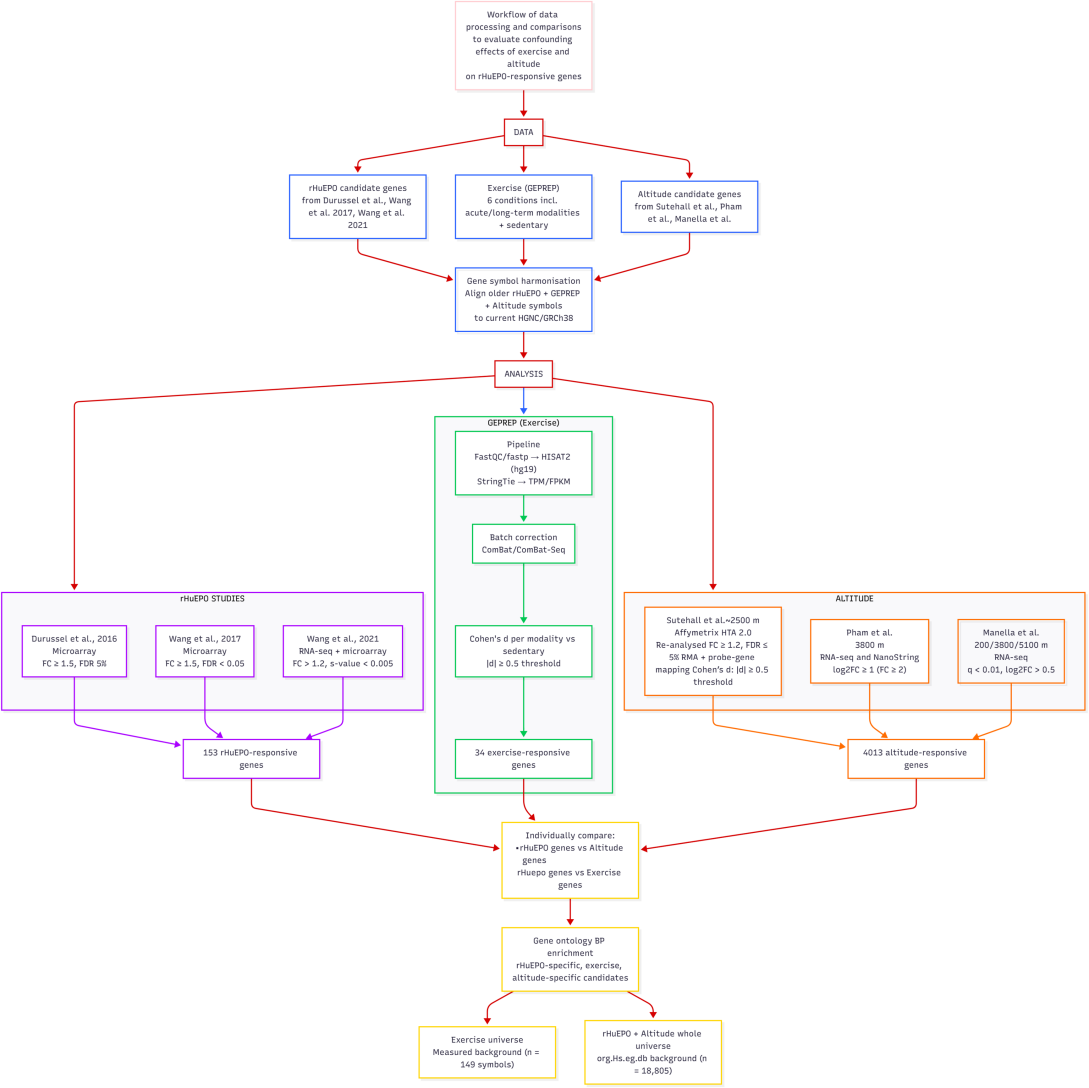
Workflow of data processing and comparisons.

We compiled 153 candidate rHuEPO‐responsive genes from Durussel et al. [11], Wang et al. [13, 14]. These candidates were then compared with standardised RNA‐seq data from GEPREP to identify which genes overlapped with the exercise‐responsive genes. To evaluate physiological confounding by hypoxia, we additionally examined their overlap with whole‐blood signatures from four independent altitude studies (Sutehall et al. [15], Pham et al. [16], Sharma and Sethy [17] and Manella et al. [18]). This analysis allowed us to distinguish genes shared with exercise or altitude exposure from those that are unique to rHuEPO administration (Figure 1).

## 2 Methods

### 2.1 Study Overview

This study investigated whether genes previously reported as responsive to rHuEPO are also altered by common physiological stressors such as exercise and altitude exposure. A total of 153 candidate genes were compiled from three transcriptomic studies of rHuEPO administration [11, 13, 14] (Table S1).

These candidate genes were evaluated in two settings:

1. Exercise datasets—Candidate genes were mapped onto uniformly reprocessed whole‐blood RNA‐seq data from the GEPREP database, enabling systematic assessment across multiple acute and long‐term exercise modalities in healthy individuals (Table S3).
2. Altitude datasets—Hypoxia‐induced responses were characterised using four high‐altitude transcriptomic studies (Sutehall et al. [15], Pham et al. [16], Sharma and Sethy [17] and Manella et al. [18]) (Tables 1 and S2). Raw data from Sutehall et al. [15] were reanalysed. From Pham et al. [16] and Manella et al. [18], we used the authors' normalised expression data and replicated their reported fold change and statistical thresholds in R to reproduce the published DEG lists and published differential expression results. Sharma and Sethy [17] reported pathway‐level IPA results without providing an underlying DEG list; this study was therefore reviewed qualitatively but excluded from the overlap analyses (Table S7).

**TABLE 1:** Summary of altitude transcriptomic datasets.

Study	Altitude (m)	Platform	Participants	Cut‐off
Sutehall et al. [15]	~2500	Affymetrix HTA 2.0 microarray	Well‐trained participants	FC ≥ 1.2, FDR ≤ 5%
Pham et al. [16]	3800	RNA‐seq and NanoString inflammatory panel	Healthy sea‐level residents	log_2_FC ≥ 1 (FC ≥ 2)
Sharma and Sethy^a^ [17]	5600, 5100	Microarray analysed with IPA (QIAGEN)	Lowlanders	*z* score > 2.5, log*p* ≥ 2.5
Manella et al. [18]	200, 3800, 5100	RNA‐seq	Lowlanders	*q* < 0.01, log_2_FC > 0.5

*Note:* This table summarises the altitude transcriptomic studies used in the present work, including the reported altitude exposure, assay platform, participant characteristics and the study‐specific differential expression cut‐off applied by each author/source. *Altitude* is reported in metres above sea level (m). *Platform* indicates the transcriptomic technology and/or analysis approach used in the original study. *Participants* describes the study population as reported. *Cut‐off* lists the author‐defined threshold(s) used to classify genes as differentially expressed (e.g., fold change, log_2_ fold change, false discovery rate, *q* value and *z* score/log*p*).

Abbreviations: FC = fold change; FDR = false discovery rate; log_2_FC = log_2_ fold change; *q* = adjusted *p* value (as reported).

^a^ Sharma and Sethy were excluded from the primary overlap analysis because no raw data or differentially expressed gene (DEG) list was available.

### 2.2 Software Environment

Analyses were conducted in R v4.3.1 using clusterProfiler v4.10.0 [20], enrichplot v1.22.0, org.Hs.eg.db v3.18.0 [21], HGNChelper v1.0.4 [22], oligo v1.62.1, arrayQualityMetrics v3.54.0 and sva v3.50.0. Affymetrix HTA 2.0 probe annotation used hta20transcriptcluster.db v8.8.0. Supporting packages included dplyr, stringr, tibble, purrr, openxlsx and ggplot2 (all CRAN).

### 2.3 Gene Symbol Harmonisation

Public datasets in GEPREP were aligned to GRCh37/hg19 (2013), and earlier rHuEPO studies (Durussel et al. [11] and Wang et al. [13]) reported genes using outdated nomenclature. To ensure comparability, we performed a systematic post hoc harmonisation of all identifiers to current HGNC‐approved symbols (GRCh38, accessed in 2025) (Table S1).

Input gene names were standardised by trimming whitespace, harmonising case and flagging provisional LOC identifiers and historical aliases. Symbols were then mapped to HGNC nomenclature via Ensembl/biomaRt (hsapiens_gene_ensembl), querying both the official symbol and synonym fields to resolve deprecated entries. For each query, a single best match was retained, prioritising an HGNC‐approved symbol with protein‐coding biotype and informative description. For all retained matches, we recorded the Ensembl gene ID, Entrez Gene ID, gene biotype and functional description.

Entries that remained unresolved, particularly LOC placeholders, were further interrogated via NCBI Gene using rentrez. Where NCBI returned a non‐LOC official symbol, the identifier was requeried in Ensembl to populate all database fields; items persisting as LOC after both passes were flagged as unmapped.

This harmonisation aligned GEPREP and older rHuEPO gene lists (hg19 based) within a single HGNC/GRCh38 annotation framework.

### 2.4 Exercise Gene Expression (GEPREP‐Based Analysis)

Only datasets involving human participants were included. Gene expression was evaluated across six exercise conditions: acute aerobic and anaerobic exercise, long‐term resistance and long‐term aerobic exercise (AE), other modalities (e.g., high‐intensity interval training [HIIT]) and a sedentary control.

All preprocessing was conducted by the GEPREP pipeline [19]. Sequencing reads underwent quality control with FastQC [23], adapter trimming with Fastp [24], alignment to the GRCh37/hg19 genome using HISAT2 [25] and transcript quantification with StringTie [26]. Expression values were reported as transcripts per million (TPM) and fragments per kilobase per million (FPKM). To correct for study‐level batch effects, GEPREP applies the ComBat and ComBat‐Seq algorithms [27, 28], and each transcript is indexed by its official gene symbol.

For the present analysis, we retrieved gene‐level expression values and evaluated their biological relevance using effect sizes.

According to Sun et al. [19], GEPREP implements RNA‐seq specific count models (Poisson/negative binomial) internally. For end users, results are presented as boxplots with KW‐derived *p* values. The precise model outputs (dispersion, size factors and GLM coefficients) are not accessible.

Specifically, Cohen's *d* was calculated in R to compare each exercise modality against the sedentary control. Genes with |*d*| ≥ 0.5 were considered to exhibit biologically meaningful responses, irrespective of nominal statistical significance, using a moderate effect‐size threshold to capture physiologically relevant changes (Table S3).

In some cases, GEPREP failed to calculate valid *p* values (not a number [NaN]), likely due to uniform expression within certain groups (zero variance). For these genes, statistical variance was insufficient for hypothesis testing, but effect sizes were still computed from group means and standard deviations so that they could be evaluated using the same Cohen's *d* criterion.

### 2.5 Altitude Gene Expression

We reanalysed whole‐blood gene expression datasets from four independent high‐altitude studies. The altitude protocols, platforms, participants and cut‐off criteria are summarised in Table 1.

For three studies (Sutehall et al. [15], Pham et al. [16] and Manella et al. [18]), raw or supplementary datasets were available (Tables [Link], [Link]). We used the authors' normalised expression data and applied the same statistical cut‐offs, fold change in R software to reproduce the published lists of DEGs.

Raw whole‐blood transcriptomic data from Sutehall et al. [15] were downloaded from the Supporting Information and reanalysed. Raw Affymetrix HTA 2.0 CEL files were processed in R using the oligo package. Quality control was performed on raw arrays using arrayQualityMetrics. Robust multiarray average (RMA) was applied for background correction, quantile normalisation and summarisation to the transcript‐cluster level. To obtain gene‐level values, probe sets were restricted to ‘main’ transcript clusters with getMainProbes. Probe to gene annotation was performed using the hta20transcriptcluster.db database, retaining unique, non‐ambiguous mappings with valid HGNC symbols. Probe sets with multiple gene assignments or missing symbols were excluded, resulting in a strictly one‐to‐one probe gene mapping.

The resulting expression matrix was aligned with phenotype metadata to ensure sample consistency. Where applicable, batch effects were addressed with ComBat (sva package) using status and subject as covariates. Additional postnormalisation QC included principal component analysis (PCA) and correlation‐based clustering to assess the sample.

### 2.6 EPO‐Responsive Genes (rHuEPO Studies)

A total of 153 genes (Table S1) previously identified as transcriptionally responsive to rHuEPO were selected based on three key transcriptomic studies [11, 13, 14] (Table 2).

**TABLE 2:** Summary of rHuEPO transcriptomic datasets and study‐specific DEG thresholds.

Study	Intervention	Platform(s)	Criteria
Durussel et al. [11]	50 IU·kg^−1^,	Microarray	FC ≥ 1.5, FDR 5%
Wang et al. [13]	20–40 IU·kg^−1^	Microarray	FC ≥ 1.5, FDR < 0.05
Wang et al. [14]	Samples from Durussel et al. [11]	RNA‐seq, microarray	FC > 1.2, *s* value < 0.005

*Note:* This table summarises the rHuEPO administration studies included in the present analysis, reporting the intervention dose, transcriptomic platform(s) and the author‐defined criteria used to identify differentially expressed genes (DEGs). Where multiple platforms were used, criteria are reported as stated in the original publication.

Abbreviations: FC = fold change; FDR = false discovery rate; rHuEPO = recombinant human erythropoietin.

The 153 EPO‐responsive genes were queried against the GEPREP database to evaluate their expression patterns across multiple exercise modalities. Genes exhibiting statistically significant changes *p* < 0.05 or returning NaN *p* values (Table 3) were further assessed for biological relevance using Cohen's *d* effect size (Table 4). A threshold of *d* ≥ 0.5 was applied to identify moderate magnitudes, thereby highlighting biologically meaningful shifts beyond statistical significance.

**TABLE 3:** GEPREP normalised expression values across exercise modalities and inactivity (genes with *p* < 0.05 or NaN).

Gene name	Acute aerobic	Acute anaerobic	Long‐term aerobic	Long‐term resistance	Other exercise	Inactivity	*p*
GYPB	1.78 ± 2.60	0.00 ± 0.00	0.00 ± 0.00	0.00 ± 0.01	0.00 ± 0.00	0.22 ± 1.06	1.18 × 10^−38^
CTSE	0.50 ± 0.75	0.00 ± 0.01	0.00 ± 0.01	0.00 ± 0.01	0.00 ± 0.00	0.07 ± 0.33	3.10 × 10^−30^
RHAG	0.32 ± 0.57	0.00 ± 0.01	0.00 ± 0.01	0.00 ± 0.01	0.00 ± 0.00	0.04 ± 0.23	4.34 × 10^−30^
SLC4A1	2.37 ± 3.18	0.01 ± 0.03	0.01 ± 0.02	0.06 ± 0.09	0.06 ± 0.16	0.41 ± 1.41	1.40 × 10^−20^
ALAS2	3.36 ± 4.32	0.06 ± 0.10	0.03 ± 0.11	0.14 ± 0.21	0.19 ± 0.43	0.72 ± 1.97	1.94 × 10^−19^
TRIM58	1.76 ± 2.37	0.02 ± 0.03	0.06 ± 0.13	0.07 ± 0.11	0.05 ± 0.10	0.36 ± 1.09	6.22 × 10^−16^
HBA1	5.52 ± 5.01	4.52 ± 1.50	2.54 ± 1.28	1.99 ± 2.00	2.95 ± 2.91	3.29 ± 3.05	3.17 × 10^−15^
SLC6A10P	0.26 ± 0.37	0.10 ± 0.14	0.06 ± 0.09	0.54 ± 0.65	0.19 ± 0.23	0.24 ± 0.43	5.55 × 10^−15^
CA1	2.69 ± 3.79	0.02 ± 0.05	0.00 ± 0.00	0.03 ± 0.09	0.01 ± 0.04	0.37 ± 1.55	6.57 × 10^−14^
CENPE	0.26 ± 0.40	0.03 ± 0.06	0.01 ± 0.04	0.05 ± 0.05	0.03 ± 0.07	0.05 ± 0.15	8.93 × 10^−14^
TFR2	0.50 ± 0.75	0.00 ± 0.01	0.14 ± 0.34	0.02 ± 0.03	0.10 ± 0.32	0.12 ± 0.39	9.86 × 10^−13^
FEM1A	7.21 ± 0.20	7.20 ± 0.26	7.16 ± 0.22	7.15 ± 0.22	7.10 ± 0.25	7.27 ± 0.23	3.38 × 10^−12^
GATA1	1.49 ± 2.08	0.01 ± 0.03	0.48 ± 1.08	0.01 ± 0.02	0.39 ± 1.02	0.32 ± 1.05	4.33 × 10^−12^
KRT1	1.58 ± 2.26	0.05 ± 0.20	0.03 ± 0.15	0.02 ± 0.03	0.09 ± 0.55	0.26 ± 0.98	4.38 × 10^−12^
PICALM	5.21 ± 0.44	5.11 ± 0.48	5.06 ± 0.55	5.15 ± 0.37	5.24 ± 0.46	4.96 ± 0.50	3.82 × 10^−11^
HBD	1.59 ± 2.12	0.13 ± 0.35	0.19 ± 0.47	0.28 ± 0.41	0.47 ± 1.28	0.55 ± 1.27	4.52 × 10^−10^
STK11	4.37 ± 0.28	4.39 ± 0.27	4.36 ± 0.29	4.32 ± 0.24	4.27 ± 0.29	4.43 ± 0.27	6.06 × 10^−7^
EPB42	1.71 ± 2.38	0.08 ± 0.06	0.35 ± 0.47	0.16 ± 0.09	0.28 ± 0.52	0.39 ± 1.04	1.05 × 10^−6^
KIF18A	0.27 ± 0.42	0.05 ± 0.06	0.03 ± 0.07	0.06 ± 0.05	0.05 ± 0.07	0.07 ± 0.14	1.14 × 10^−6^
ATP6V1D	4.06 ± 0.23	4.04 ± 0.24	4.08 ± 0.24	4.08 ± 0.21	4.12 ± 0.22	4.01 ± 0.23	1.32 × 10^−5^
TPM1	11.19 ± 0.50	11.40 ± 0.49	11.37 ± 0.44	11.48 ± 0.50	11.40 ± 0.42	11.34 ± 0.47	1.47 × 10^−5^
ARF1	6.34 ± 0.22	6.35 ± 0.23	6.30 ± 0.21	6.23 ± 0.20	6.25 ± 0.21	6.34 ± 0.21	1.48 × 10^−5^
SH3GL1	2.69 ± 0.64	2.70 ± 0.52	2.59 ± 0.64	2.63 ± 0.66	2.70 ± 0.69	2.49 ± 0.62	5.10 × 10^−5^
GYPE	0.62 ± 1.11	0.05 ± 0.07	0.04 ± 0.06	0.07 ± 0.07	0.07 ± 0.10	0.14 ± 0.46	1.04 × 10^−4^
WDTC1	4.41 ± 0.24	4.42 ± 0.25	4.44 ± 0.22	4.38 ± 0.24	4.37 ± 0.29	4.47 ± 0.24	1.20 × 10^−4^
FBXO30	1.71 ± 0.32	1.63 ± 0.33	1.63 ± 0.38	1.71 ± 0.28	1.65 ± 0.37	1.60 ± 0.33	1.21 × 10^−4^
KIF23	0.18 ± 0.32	0.05 ± 0.10	0.15 ± 0.29	0.08 ± 0.09	0.14 ± 0.29	0.08 ± 0.19	1.35 × 10^−4^
FBXO9	4.02 ± 0.55	3.98 ± 0.64	3.99 ± 0.63	4.06 ± 0.51	3.88 ± 0.98	4.15 ± 0.56	7.06 × 10^−4^
UBQLN1	4.42 ± 0.24	4.42 ± 0.26	4.35 ± 0.23	4.36 ± 0.19	4.36 ± 0.29	4.35 ± 0.25	1.67 × 10–3
ANKRD9	5.54 ± 0.42	5.54 ± 0.47	5.42 ± 0.37	5.43 ± 0.43	5.41 ± 0.51	5.43 ± 0.41	2.26 × 10^−3^
NEDD4L	2.74 ± 0.47	2.68 ± 0.46	2.61 ± 0.46	2.79 ± 0.50	2.81 ± 0.44	2.81 ± 0.48	2.46 × 10^−3^
RBL1	1.81 ± 0.29	1.75 ± 0.34	1.81 ± 0.29	1.88 ± 0.27	1.76 ± 0.31	1.86 ± 0.30	2.72 × 10^−3^
GMPR	6.24 ± 0.26	6.27 ± 0.31	6.25 ± 0.29	6.18 ± 0.24	6.21 ± 0.36	6.26 ± 0.26	4.15 × 10^−3^
ABCG2	0.11 ± 0.13	0.12 ± 0.15	0.13 ± 0.13	0.17 ± 0.13	0.15 ± 0.16	0.12 ± 0.12	6.59 × 10^−3^
PSME4	3.94 ± 0.28	3.93 ± 0.32	3.88 ± 0.29	4.02 ± 0.27	3.91 ± 0.27	3.89 ± 0.31	8.00 × 10^−3^
FBXL4	1.70 ± 0.31	1.68 ± 0.35	1.68 ± 0.34	1.82 ± 0.31	1.70 ± 0.35	1.75 ± 0.34	1.29 × 10^−2^
SKI	2.69 ± 0.52	2.60 ± 0.55	2.58 ± 0.54	2.76 ± 0.46	2.62 ± 0.56	2.56 ± 0.55	1.37 × 10^−2^
EPN1	5.24 ± 0.29	5.27 ± 0.24	5.25 ± 0.26	5.26 ± 0.27	5.19 ± 0.30	5.29 ± 0.25	1.96 × 10^−2^
ISCA1	4.81 ± 0.33	4.83 ± 0.35	4.82 ± 0.37	4.90 ± 0.30	4.81 ± 0.37	4.77 ± 0.34	2.66 × 10^−2^
AQP1	0.00 ± 0.00	0.01 ± 0.03	0.00 ± 0.02	0.00 ± 0.01	0.00 ± 0.01	0.00 ± 0.03	2.91 × 10^−2^
AP2B1	3.90 ± 0.30	3.87 ± 0.29	3.91 ± 0.30	3.89 ± 0.27	3.96 ± 0.27	3.87 ± 0.27	3.84 × 10^−2^
HBB	9.71 ± 1.81	10.10 ± 1.84	8.96 ± 2.56	9.48 ± 2.10	9.94 ± 1.98	9.72 ± 1.92	NaN
UBA52	8.42 ± 1.06	8.64 ± 0.70	8.60 ± 0.55	8.43 ± 0.82	8.24 ± 1.42	8.39 ± 1.03	NaN
SLC25A37	7.55 ± 1.43	7.15 ± 1.50	7.08 ± 1.42	7.34 ± 1.26	7.15 ± 1.66	7.23 ± 1.52	NaN
SLC25A39	7.35 ± 1.95	6.94 ± 1.82	7.43 ± 1.14	7.65 ± 1.44	7.67 ± 1.60	7.43 ± 1.47	NaN
BLVRB	6.33 ± 1.26	6.12 ± 1.34	6.16 ± 1.70	6.07 ± 1.21	6.27 ± 1.46	6.06 ± 1.57	NaN
ATP6V0C	6.15 ± 0.94	6.24 ± 1.07	6.14 ± 1.20	5.48 ± 1.44	5.99 ± 1.13	6.15 ± 0.98	NaN
CTSB	5.50 ± 0.61	5.53 ± 0.52	5.49 ± 0.59	5.53 ± 0.54	5.41 ± 0.62	5.43 ± 0.59	NaN
PCGF5	5.00 ± 1.50	5.02 ± 1.41	4.73 ± 1.64	5.45 ± 1.22	5.33 ± 1.18	4.80 ± 1.51	NaN
STRADB	4.86 ± 1.12	4.95 ± 1.12	4.61 ± 1.54	4.97 ± 0.99	4.76 ± 1.30	5.03 ± 1.08	NaN
PSMF1	4.93 ± 0.71	4.99 ± 0.64	5.06 ± 0.57	4.90 ± 0.69	5.03 ± 0.66	4.98 ± 0.74	NaN
FBXO7	5.00 ± 1.24	4.74 ± 1.36	5.19 ± 1.13	4.69 ± 1.38	4.99 ± 1.17	4.71 ± 1.46	NaN
BCL2L1	4.67 ± 1.55	4.55 ± 1.39	4.65 ± 1.32	4.48 ± 1.43	4.68 ± 1.66	3.99 ± 1.78	NaN
LGALS3	4.04 ± 1.36	4.11 ± 1.21	4.14 ± 1.41	4.33 ± 1.21	4.40 ± 1.10	4.13 ± 1.21	NaN
TFRC	3.95 ± 0.84	3.89 ± 0.98	3.86 ± 0.89	3.77 ± 0.68	3.83 ± 0.77	4.00 ± 0.82	NaN
FECH	3.33 ± 0.94	3.45 ± 0.85	3.34 ± 0.89	3.69 ± 0.53	3.45 ± 0.74	3.47 ± 0.78	NaN
FCHO2	2.15 ± 0.71	2.10 ± 0.72	2.05 ± 0.89	2.16 ± 0.63	2.17 ± 0.81	2.05 ± 0.87	NaN
CD3G	1.49 ± 1.16	1.34 ± 1.14	1.09 ± 1.33	1.66 ± 0.82	1.22 ± 1.17	1.58 ± 0.95	NaN
TAL1	1.32 ± 1.27	1.04 ± 1.48	1.24 ± 1.59	1.73 ± 1.00	1.52 ± 1.20	0.96 ± 1.57	NaN
KLC3	0.54 ± 1.08	0.55 ± 1.16	0.08 ± 0.86	−0.23 ± 1.90	−0.56 ± 2.26	−0.03 ± 1.49	NaN
RBM38	6.24 ± 0.86	6.23 ± 0.83	6.16 ± 0.78	6.15 ± 0.74	6.10 ± 0.76	6.43 ± 0.74	NaN
SNCA	5.47 ± 0.86	2.42 ± 1.31	3.83 ± 1.56	5.51 ± 0.59	4.85 ± 0.91	4.67 ± 1.47	NaN
SELENBP1	4.36 ± 1.47	4.46 ± 1.63	4.46 ± 1.01	4.29 ± 1.08	4.19 ± 1.31	4.61 ± 1.30	NaN
BPGM	3.40 ± 1.16	3.47 ± 1.19	3.38 ± 1.44	2.84 ± 1.39	3.18 ± 1.28	3.46 ± 1.27	NaN
TENT5C	2.95 ± 1.59	2.67 ± 1.75	3.17 ± 1.61	2.80 ± 1.46	3.07 ± 1.50	3.24 ± 1.24	NaN
MARCHF8	2.64 ± 1.32	2.69 ± 1.24	2.83 ± 1.24	3.24 ± 0.78	2.69 ± 1.25	3.00 ± 1.27	NaN
SIAH2	2.92 ± 1.24	2.73 ± 1.17	2.72 ± 1.18	3.00 ± 0.96	3.14 ± 0.88	2.60 ± 1.24	NaN
OSBP2	1.45 ± 1.63	1.28 ± 1.62	1.67 ± 1.29	1.56 ± 1.53	1.10 ± 1.99	1.74 ± 1.29	NaN
E2F2	0.26 ± 0.80	0.63 ± 0.82	0.66 ± 0.49	0.76 ± 0.57	0.58 ± 0.75	0.49 ± 0.67	NaN
CDK4	3.18 ± 0.35	3.17 ± 0.35	3.16 ± 0.37	3.12 ± 0.41	3.18 ± 0.37	3.09 ± 0.36	3.13 × 10^−3^
MAPKAPK5	2.83 ± 0.26	2.83 ± 0.26	2.81 ± 0.26	2.81 ± 0.23	2.75 ± 0.27	2.86 ± 0.26	6.94 × 10^−3^
GUK1	6.94 ± 1.29	6.24 ± 2.02	6.22 ± 1.99	6.46 ± 1.86	6.99 ± 1.19	6.41 ± 1.61	NaN
CD28	0.94 ± 0.75	1.05 ± 0.53	0.76 ± 0.60	0.99 ± 0.51	0.98 ± 0.60	0.92 ± 0.57	NaN
DCTD	3.20 ± 0.33	3.25 ± 0.30	3.24 ± 0.31	3.34 ± 0.28	3.21 ± 0.37	3.24 ± 0.31	3.59 × 10^−2^
MAP4K4	3.56 ± 0.55	3.52 ± 0.51	3.54 ± 0.59	3.83 ± 0.43	3.51 ± 0.83	3.48 ± 0.59	NaN
BRD4	3.82 ± 1.05	3.74 ± 1.21	3.53 ± 1.53	3.69 ± 1.15	3.62 ± 1.38	3.43 ± 1.32	NaN
LRRC8C	1.48 ± 0.44	1.36 ± 0.48	1.44 ± 0.49	1.62 ± 0.35	1.61 ± 0.45	1.38 ± 0.43	4.26 × 10^−8^
CDK6	0.84 ± 0.51	0.82 ± 0.46	0.93 ± 0.49	1.13 ± 0.39	1.05 ± 0.46	0.81 ± 0.44	1.53 × 10^−10^
TNRC6A	2.94 ± 0.49	2.89 ± 0.51	2.85 ± 0.55	2.93 ± 0.41	2.83 ± 0.57	2.89 ± 0.47	NaN
PSENEN	3.58 ± 0.66	3.53 ± 0.89	3.64 ± 0.62	3.56 ± 0.74	3.66 ± 0.72	3.58 ± 0.73	NaN
TNRC6C	1.96 ± 0.77	1.83 ± 0.79	1.76 ± 0.85	1.87 ± 0.65	1.74 ± 0.76	1.92 ± 0.99	NaN
RPS27A	7.77 ± 1.26	7.66 ± 1.30	7.79 ± 0.89	7.54 ± 1.22	7.61 ± 1.27	7.74 ± 1.14	NaN
JUN	5.16 ± 0.81	5.20 ± 0.59	5.01 ± 0.70	4.95 ± 1.01	4.95 ± 1.01	5.04 ± 0.79	NaN
S1PR1	3.29 ± 0.68	3.27 ± 0.71	3.29 ± 0.63	3.52 ± 0.52	3.42 ± 0.68	2.97 ± 0.73	NaN
DMTN	5.39 ± 1.22	5.24 ± 1.47	5.25 ± 1.64	5.51 ± 1.26	5.28 ± 1.46	5.30 ± 1.47	NaN
MAP2K7	3.72 ± 0.32	3.74 ± 0.30	3.73 ± 0.29	3.69 ± 0.33	3.65 ± 0.32	3.81 ± 0.30	1.26 × 10^−6^
PHC1	1.30 ± 0.35	1.27 ± 0.38	1.29 ± 0.37	1.49 ± 0.30	1.30 ± 0.39	1.32 ± 0.38	2.08 × 10^−4^
ADD1	5.32 ± 0.28	5.34 ± 0.28	5.33 ± 0.24	5.29 ± 0.25	5.26 ± 0.29	5.40 ± 0.27	3.33 × 10^−6^
FKBP8	7.20 ± 1.41	7.07 ± 1.37	7.25 ± 1.13	6.92 ± 1.70	7.17 ± 1.14	7.36 ± 1.43	NaN
SLC6A8	5.11 ± 0.31	5.09 ± 0.33	5.03 ± 0.33	4.96 ± 0.28	5.07 ± 0.33	4.95 ± 0.34	2.55 × 10^−9^
MAPKAPK2	5.48 ± 0.23	5.47 ± 0.27	5.41 ± 0.25	5.41 ± 0.24	5.42 ± 0.28	5.42 ± 0.28	3.63 × 10^−2^
UBB	9.14 ± 1.00	9.19 ± 1.05	9.10 ± 1.22	8.95 ± 1.05	8.75 ± 1.43	9.27 ± 1.03	NaN
IFI27	3.99 ± 0.96	3.77 ± 1.23	4.29 ± 0.78	4.20 ± 0.77	4.30 ± 0.87	3.82 ± 1.10	NaN
RAP1GAP	0.46 ± 0.99	0.01 ± 0.04	0.07 ± 0.17	0.02 ± 0.03	0.04 ± 0.12	0.09 ± 0.43	2.96 × 10^−8^
YOD1	2.03 ± 1.44	1.57 ± 1.79	2.23 ± 1.25	1.84 ± 1.64	1.62 ± 1.66	1.74 ± 1.62	NaN
CCR7	2.16 ± 2.88	0.08 ± 0.12	0.31 ± 0.61	0.09 ± 0.10	0.27 ± 0.46	0.57 ± 1.59	8.90 × 10^−4^
EEF1D	7.83 ± 0.53	7.77 ± 0.60	7.78 ± 0.5	7.65 ± 0.48	7.74 ± 0.68	7.86 ± 0.52	2.95 × 10^−3^
TNS1	4.96 ± 0.45	4.95 ± 0.60	4.93 ± 0.46	4.80 ± 0.49	4.82 ± 0.69	4.89 ± 0.50	2.32 × 10^−2^
ADIPOR1	5.99 ± 1.28	5.97 ± 1.31	6.05 ± 1.28	6.15 ± 1.20	5.98 ± 1.34	6.15 ± 1.30	NaN
UBXN6	5.63 ± 0.91	5.64 ± 0.96	5.64 ± 0.77	5.65 ± 1.10	5.61 ± 1.06	5.70 ± 0.82	NaN
MIF	4.20 ± 0.78	4.24 ± 0.63	4.20 ± 0.69	4.30 ± 0.61	4.18 ± 0.98	4.17 ± 0.75	NaN
CD247	2.31 ± 1.50	2.23 ± 1.77	2.41 ± 1.56	2.79 ± 0.89	2.94 ± 1.20	2.47 ± 1.46	NaN
LEF1	2.53 ± 1.46	3.37 ± 0.75	2.56 ± 1.16	3.08 ± 1.05	1.76 ± 1.33	2.61 ± 1.31	NaN
SKAP1	1.19 ± 1.33	1.01 ± 1.63	1.39 ± 1.73	1.63 ± 0.87	1.50 ± 1.10	1.33 ± 1.22	NaN

*Note:* Normalised expression values as provided by GEPREP [19]. The table displays only genes with *p* < 0.05 or with noncalculable *p* values (NaN). *p* values reflect comparisons of each exercise modality against inactivity, as reported by GEPREP. The exact normalisation and any transformation applied by GEPREP are not publicly documented.

**TABLE 4:** Cohen's *d* effect sizes for exercise modalities versus inactivity.

Gene name	Acute aerobic Cohen_d	Acute anaerobic Cohen_d	Long‐term aerobic Cohen_d	Long‐term resistance Cohen_d	Other exercise Cohen_d
ADD1	−0.29	−0.21	−0.27	−0.42	−0.50
ALAS2	0.78	−0.47	−0.49	−0.41	−0.37
ARF1	0.00	0.04	−0.19	−0.53	−0.42
ATP6V0C	0.00	0.08	−0.00	−0.54	−0.15
CA1	0.80	−0.31	−0.33	−0.30	−0.32
CCR7	0.68	−0.43	−0.21	−0.42	−0.25
CD3D	0.60	−0.44	0.01	−0.40	−0.08
CDK6	0.06	0.02	0.25	0.77	0.53
CENPE	0.69	−0.17	−0.36	0.00	−0.17
CTSE	0.74	−0.30	−0.30	−0.30	−0.30
EPB42	0.71	−0.42	−0.05	−0.31	−0.13
FEM1A	−0.27	−0.28	−0.48	−0.53	−0.70
GATA1	0.71	−0.41	0.15	−0.41	0.06
GYPB	0.78	−0.29	−0.29	−0.29	−0.29
GYPE	0.56	−0.27	−0.30	−0.21	−0.21
HBA1	0.53	0.51	−0.32	−0.50	−0.11
HBD	0.59	−0.45	−0.37	−0.28	−0.06
KIF18A	0.63	−0.18	−0.36	−0.09	−0.18
KRT1	0.75	−0.29	−0.32	−0.34	−0.21
LEF1	−0.05	0.71	−0.04	0.39	−0.64
LRRC8C	0.23	−0.04	0.13	0.61	0.52
MAP2K7	−0.29	−0.23	−0.27	−0.38	−0.51
MAP4K4	0.14	0.07	0.10	0.67	0.04
PICALM	0.53	0.30	0.19	0.43	0.58
RHAG	0.64	−0.24	−0.24	−0.24	−0.24
S1PR1	0.45	0.41	0.46	0.86	0.63
SIAH2	0.25	0.10	0.09	0.36	0.50
SLC4A1	0.79	−0.40	−0.40	−0.35	−0.34
SLC6A10P	0.05	−0.43	−0.57	0.54	−0.14
SNCA	0.66	−1.61	−0.55	0.75	0.14
STK11	−0.21	−0.14	−0.25	−0.43	−0.57
TAL1	0.25	0.05	0.17	0.58	0.40
TFR2	0.63	−0.43	0.05	−0.36	−0.05
TRIM58	0.75	−0.44	−0.38	−0.37	−0.40

*Note:* Cohen's *d* values were calculated using the normalised expression values provided by GEPREP [19]. The exact normalisation and any transformation applied by GEPREP are not publicly documented. The table displays only genes with *p* < 0.05 or with noncalculable *p* values (NaN) in the corresponding GEPREP modality‐versus‐inactivity comparisons. Negative Cohen's *d* indicates lower expression in the exercise modality relative to inactivity, and positive Cohen's *d* indicates higher expression relative to inactivity.

Because these studies used different sequencing platforms (microarray, RNA‐seq and IPA), exposure durations, participant characteristics and statistical thresholds for defining DEGs, the resulting gene lists were not fully harmonised. These methodological heterogeneities are inherent to cross‐study comparisons.

### 2.7 Two‐Step Overlap Framework

To ensure a reproducible and balanced comparison, we applied a two‐step strategy. Firstly, a comprehensive hypoxia‐responsive panel was compiled by aggregating unique DEGs across the reproducible altitude studies [15, 16, 18] (Table S2). Second, each altitude and exercise gene set was individually compared with the EPO‐responsive genes to identify overlaps (Table S8).

### 2.8 Functional Enrichment Analysis

Gene Ontology (GO) enrichment analysis of biological processes (BPs) was conducted using the clusterProfiler R package [20], with annotations from org.Hs.eg.db [21]. Analyses were performed across three contexts, rHuEPO, exercise, and altitude, with attention to appropriate background universes and uniform preprocessing to ensure replicability and comparability.

### 2.9 Background Universes

For exercise‐responsive genes, the enrichment universe was restricted to the set of DEGs in the exercise dataset (*n* = 149). This measured background (147 unique Entrez IDs postmapping) reflects the actual testable gene space, minimising inflation of enrichment significance.

For the rHuEPO and altitude datasets, only DEG lists were available. Consequently, the full human genome annotated in org.Hs.eg.db (*n* = 18,805) was used as the enrichment universe. While genome‐wide backgrounds can overestimate significance compared with measured universes, this approach was applied consistently when measured data were unavailable, ensuring transparency and comparability.

### 2.10 GO rHuEPO Dataset

Candidate genes were compiled from three independent rHuEPO administration studies [11, 13, 14], chosen for their consistent design and gene expression endpoints. Each gene list was analysed individually and as part of a combined rHuEPO‐specific panel (Table 2), intended to capture both shared and condition‐specific effects. Functional categories were visualised via network‐style cnetplots (Table S9 and Figures 3 and 4).

### 2.11 GO Exercise Dataset

Exercise candidate genes were selected based on prior differential expression and biological relevance. Enrichment was evaluated using a measured universe of 149 detectable genes. Mapping and results are in Table S9, with top terms visualised in Figures 5 and 6.

### 2.12 GO Altitude Dataset

Altitude‐responsive genes were derived from transcriptomic profiling in three studies of high‐altitude exposure. To isolate altitude‐specific effects, overlapping genes with rHuEPO and exercise sets were removed in the ‘altitude‐unique’ subset (Table S8). Due to the absence of a quantifiable gene universe, the full genome was used as background. Enrichment results (Table S9) were visualised with both bar style and network cnetplots (Figures 7 and 8).

## 3 Results

### 3.1 EPO‐Specific Genes

Fifty EPO‐responsive genes did not overlap with genes from exercise or altitude exposure. These transcripts were not altered by any exercise modality in the GEPREP dataset, with no statistically significant differences and Cohen's *d* values below the 0.5 biological relevance threshold. They were also absent from all compiled altitude‐responsive gene sets. This stability across diverse physiological conditions suggests minimal susceptibility to environmental or training‐induced factors. Their exclusive regulation in rHuEPO datasets positions these 50/153 genes as the strongest candidates for high specificity transcriptomic biomarkers in antidoping applications (Tables 5 and S1).

**TABLE 5:** List of the 50 unique rHuEPO‐responsive genes.

AK2	DCAF10	FCHO2	MAPKAPK5	RBBP4
ALAD	DCAF11	ITSN1	MIF	RBL1
AP2B1	DCTD	JUN	MRS2	SKAP1
ATP6V0A1	DCTN4	KIF15	NEDD4L	SKI
BTRC	DCUN1D1	LOC286444 (NCBI RPS2P55)	NR3C1	SKP1
CD247	DCUN1D4	LOC389599 (NCBI STRADBP1)	PHC1	TNRC6A
CD28	E2F4	LOC441455 (NCBI MKRN10P)	PIK3R1	TNRC6C
CDK4	FBXL4	LOC100130562 (NCBI RPS2P5)	PPM1A	UBA52
COPS3	FBXO30	LOC100131164	PSENEN	UBE2F
CTSB	FBXO9	MAPKAPK2	PSMF1	UBQLN1

*Note:* This table lists the 50 unique genes identified as rHuEPO‐responsive in the source studies included in the present work. NCBI‐designated pseudogenes are shown for transparency but were excluded from all downstream functional enrichment analyses.

Abbreviations: NCBI = National Center for Biotechnology Information; rHuEPO = recombinant human erythropoietin.

### 3.2 Exercise‐Responsive Genes

Of the 153 rHuEPO‐responsive candidate genes, 34 (22%) showed biologically meaningful expression changes (Cohen's *d* ≥ 0.5) in at least one exercise modality (Table 3). These exercise‐responsive genes were distributed across all six analysed conditions: acute aerobic, acute resistance, long‐term aerobic, long‐term resistance, other exercise modalities and sedentary control.

In terms of statistical testing, 56 of 153 genes (37%) showed significant differences (*p* ≤ 0.05), 50 (33%) returned NaN *p* values and 40 (26%) had *p* ≥ 0.05. Seven genes (5%) were not represented in the GEPREP database (Tables 3 and S3).

Among the 34 exercise‐responsive genes, all overlapped with the EPO‐responsive set (34/34; 100%). Of these, 25 (74%) were also shared with altitude, while the remaining 9 (26%) overlapped only with EPO. No exercise‐responsive genes were unique to exercise alone. This complete overlap underscores the strong potential for physiological cross‐reactivity, as these transcripts responded to both rHuEPO and exercise stimuli.

### 3.3 Gene Expression in Response to Altitude Exposure

Across the altitude datasets, hypoxic exposure induces substantial transcriptomic changes, though the scale and composition of DEGs varied by study design and analytical approach (Table 1).

- Sutehall et al. [15]: Identified 29 consistently regulated genes in whole blood following a 27‐day stay at ~2500 m (microarray).
- Pham et al. [16]: Reported 163 DEGs on Day 1 and 285 on Day 3 during acute exposure to 3800 m (RNA‐seq).
- Manella et al. [18]: Detected 3775 altitude‐responsive genes at different altitudes from 3800 to 5100 m over 21 days (RNA‐seq).

These three reproducible datasets gave 4013 altitude‐responsive genes (Table S2). When cross‐referenced with the 153 rHuEPO‐responsive candidates, 94 overlapping transcripts were identified (Table S8). These overlaps underscore the need for cautious interpretation of transcriptomic biomarkers, as several candidates may reflect general hypoxia‐driven responses rather than rHuEPO‐specific regulation.

Sharma and Sethy [17] reported 27 altitude‐responsive genes using IPA with a log ratio cut‐off of ±1‐fold and pathway filtering (*z* score > 2.5, −log*p* ≥ 2.5). Of these, 23 overlapped with genes already captured by other datasets, and 4 were unique but did not overlap with the rHuEPO‐responsive set. However, as no supplementary DEG list or raw data were provided, this dataset was not reproducible and was excluded from the primary overlap analysis (Tables 1 and S7). Its exclusion did not alter the study's conclusions.

The distribution of overlaps highlights that most shared signals were driven by the large Manella [18] dataset. In contrast, overlaps with Sutehall and Pham were more restricted and time dependent, suggesting that confounding effects from hypoxia vary considerably by study design and exposure duration (Table 6).

**TABLE 6:** Distribution of rHuEPO–altitude overlapping genes across studies.

Category	Gene count	Notes
Total unique genes (rHuEPO ∩ altitude)	94	Union of all three altitude studies
Unique to Manella	63	Overlap only between rHuEPO and Manella (57 at 3800 m; 6 at 5100 m)
Unique to Pham (Day 1—HA1)	0	Not present
Unique to Pham (Day 3—HA3)	4	Present only between rHuEPO Pham (HA3)
Unique to Sutehall	1	Present only between rHuEPO and Sutehall
Shared between Manella and Pham (HA3)	25	rHuEPO‐overlapping genes in both, absent in Sutehall
Shared between Manella and Sutehall	1	rHuEPO‐overlapping genes in both, absent in Pham (HA3)
Shared between Pham (HA3) and Sutehall	0	None
Shared across all three studies	0	None

*Note:* This table summarises how genes overlapping between the rHuEPO‐responsive set and altitude‐responsive datasets are distributed across individual altitude studies and their intersections. *Total unique genes (rHuEPO ∩ altitude)* represents the union of all genes overlapping rHuEPO with any included altitude dataset. ‘Unique’ categories indicate genes overlapping rHuEPO in only one altitude study. ‘Shared’ categories indicate genes overlapping rHuEPO in two or more altitude studies, with notes specifying which study intersections apply.

Abbreviations: HA1 = Pham Day 1 high‐altitude contrast; HA3 = Pham Day 3 high‐altitude contrast; rHuEPO = recombinant human erythropoietin.

### 3.4 Overlap Among EPO, Altitude and Exercise‐Responsive Genes

**FIGURE 2:**
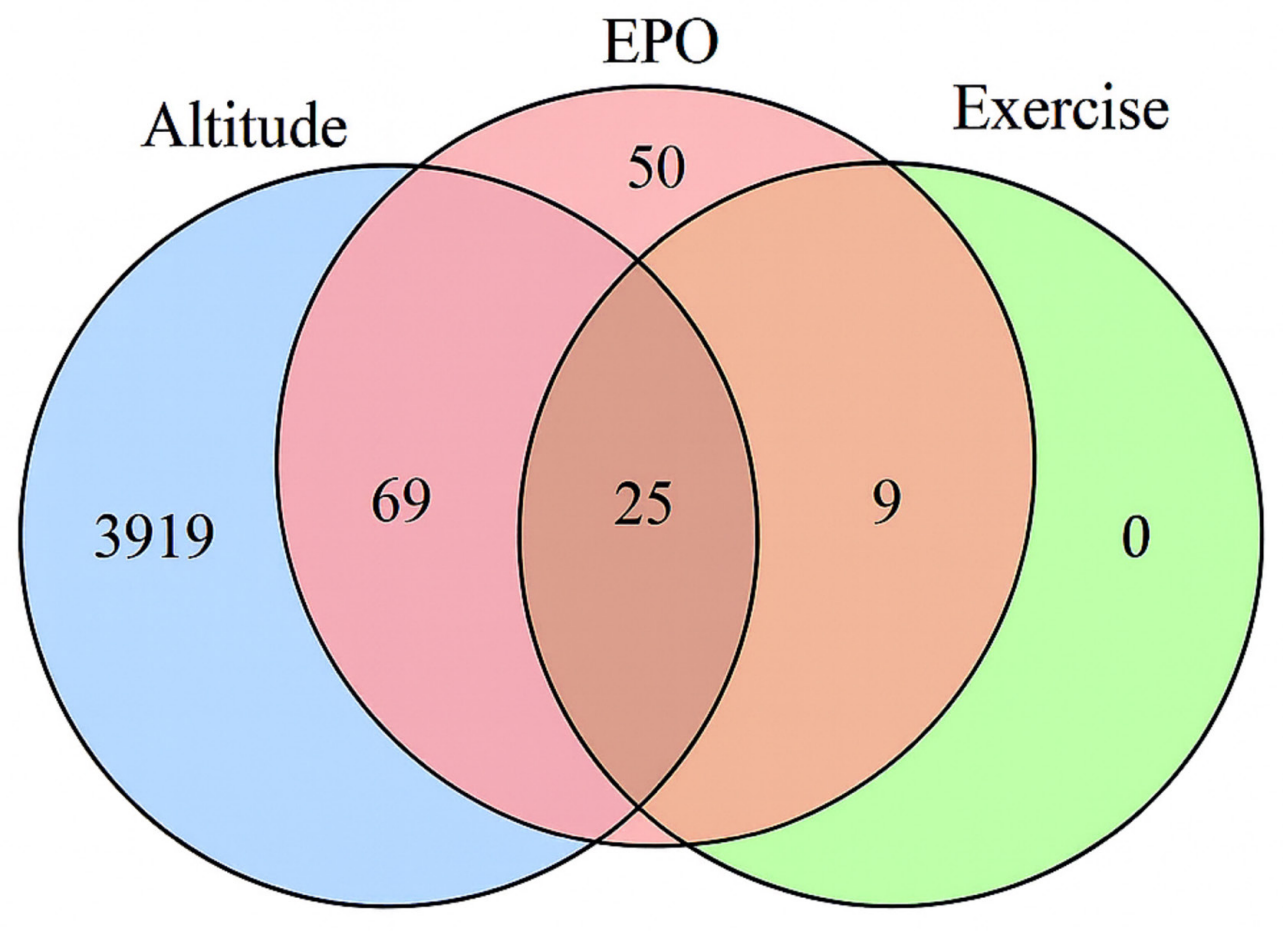
Venn diagram—Three‐way overlap among rHuEPO‐candidate genes (EPO; *n* = 153), altitude‐responsive genes (altitude; *n* = 4013; union across reproducible studies) and exercise‐responsive genes (exercise; *n* = 34, derived from within the EPO list using GEPREP). Numbers indicate genes unique to each set or shared across sets.

Across the three datasets (EPO, *n* = 153; altitude, *n* = 4013; exercise, *n* = 34), most EPO‐candidate genes overlapped with at least one physiological factor (Figure 2). All exercise‐responsive genes overlapped with the EPO list, indicating that training and pharmacological stimulation activate many of the same transcriptional signals.

Among the 34 exercise genes, 25 were shared across EPO and altitude (15 overlapped with the Manella dataset and 10 with the Pham dataset). The remaining nine genes overlapped only between exercise and EPO. Notably, no gene was shared between altitude and exercise without also overlapping with EPO.

In total, 94 altitude‐responsive genes overlapped with the 153 EPO‐responsive candidates. Most (89 genes) originated from the Manella dataset, with 63 unique to that study. Of these, 57/63 were already significant at ~3800 m, an athlete‐relevant altitude, whereas only six appeared at 5100 m. In the Pham dataset, 29 overlaps with EPO‐responsive genes were identified: four unique to Pham and 25 also shared with Manella. These shared transcripts were likewise evident at 3800 m, consistent with the altitude tested in Pham. In the Sutehall dataset, two overlaps were detected: one unique to that study and one shared with the Manella dataset.

Together, these distributions show that although rHuEPO‐responsive candidate markers can be identified, their interpretation is complicated by the overlapping transcriptional footprint of exercise and hypoxia. The predominance of overlap at ~3800 m underscores the particular relevance of moderate altitude exposure as a confounder in athlete populations (Tables 6 and S8).

### 3.5 GO Enrichment

#### 3.5.1 EPO‐Responsive Genes

GO BP analysis identified several significantly enriched pathways from 50 genes; it mapped 45 genes. The most strongly over‐represented terms were protein neddylation (GO:0045116; four genes; fold enrichment 61.1; *q* = 5.3 × 10^−4^) and the SCF‐dependent proteasomal ubiquitin‐dependent protein catabolic process (GO:0031146; four genes; fold enrichment 40.7; *q* = 1.4 × 10^−3^). Related categories included regulation of protein neddylation (GO:2000434; three genes; fold enrichment 64.1; *q* = 3.1 × 10^−3^), positive regulation of protein modification by small protein conjugation or removal (GO:1903322; five genes; fold enrichment 16.1; *q* = 3.1 × 10^−3^) and positive regulation of post‐translational protein modification (GO:1901875; five genes; fold enrichment 15.9; *q* = 3.1 × 10^−3^) (Table S9).

Additional significantly enriched categories captured broader ubiquitin‐related processes, including regulation of protein modification by small protein conjugation or removal (GO:1903320; six genes; fold enrichment 10.6; *q* = 3.4 × 10^−3^), regulation of post‐translational protein modification (GO:1901873; six genes; fold enrichment 10.3; *q* = 3.4 × 10^−3^) and protein K48‐linked ubiquitination (GO:0070936; four genes; fold enrichment 17.1; *q* = 1.1 × 10^−2^). The broader proteasome‐mediated ubiquitin‐dependent protein catabolic process (GO:0043161; seven genes; fold enrichment 6.4; *q* = 1.1 × 10^−2^) was also significantly enriched.

Outside ubiquitin‐related pathways, enrichment was detected for clathrin‐dependent endocytosis (GO:0072583; three genes; fold enrichment 26.2; *q* = 2.1 × 10^−2^), T cell receptor signalling pathway (GO:0050852; four genes; fold enrichment 11.3; *q* = 3.6 × 10^−2^) and RNA processing categories such as positive regulation of nuclear‐transcribed mRNA poly(A) tail shortening (GO:0060213; two genes; fold enrichment 65.8; *q* = 3.6 × 10^−2^) and regulation of nuclear‐transcribed mRNA poly(A) tail shortening (GO:0060211; two genes; fold enrichment 57.0; *q* = 4.4 × 10^−2^).

**FIGURE 3:**
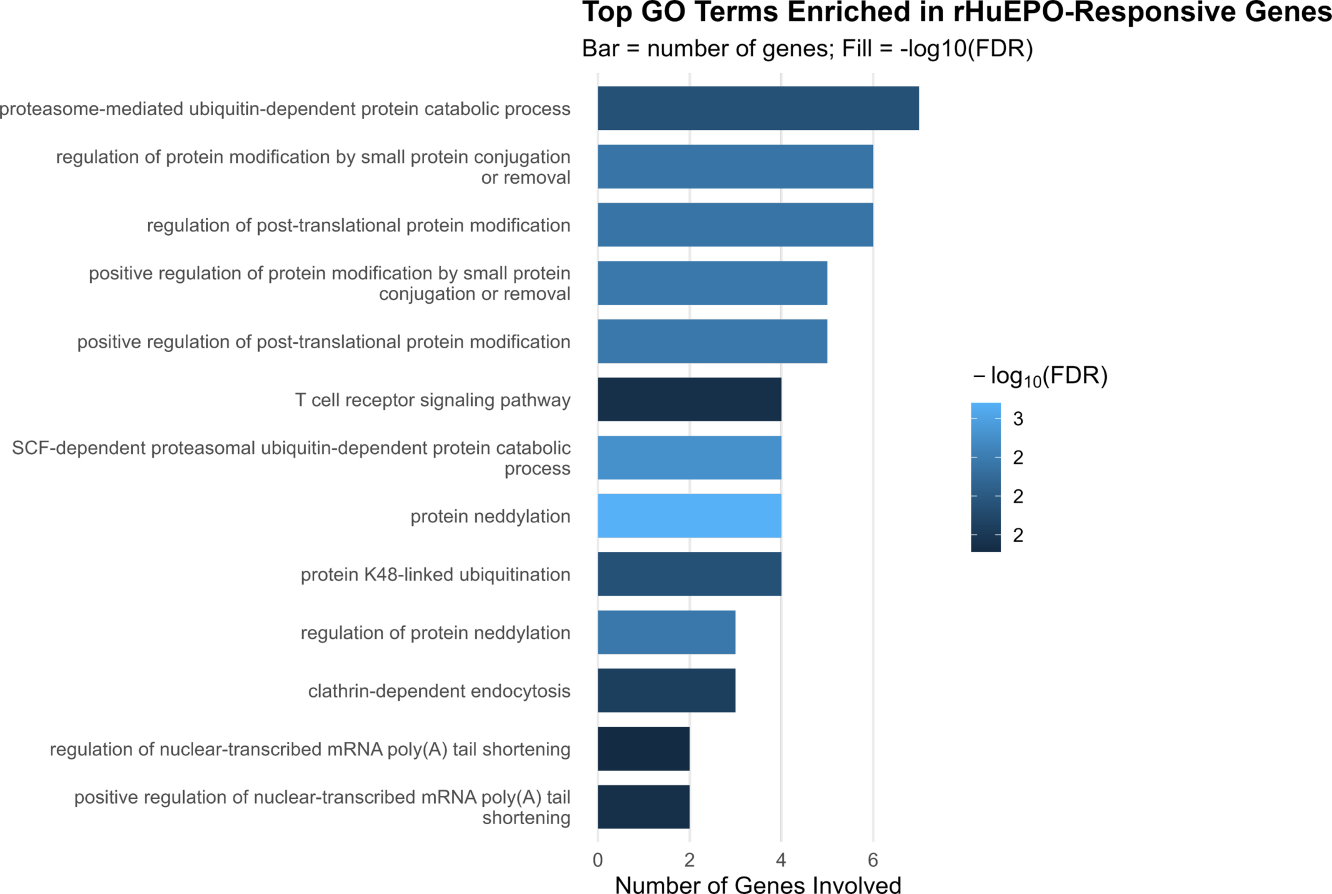
Top 13 GO biological processes enriched in rHuEPO‐responsive genes. Bars show the number of genes per term; colour encodes −log_10_(FDR).

**FIGURE 4:**
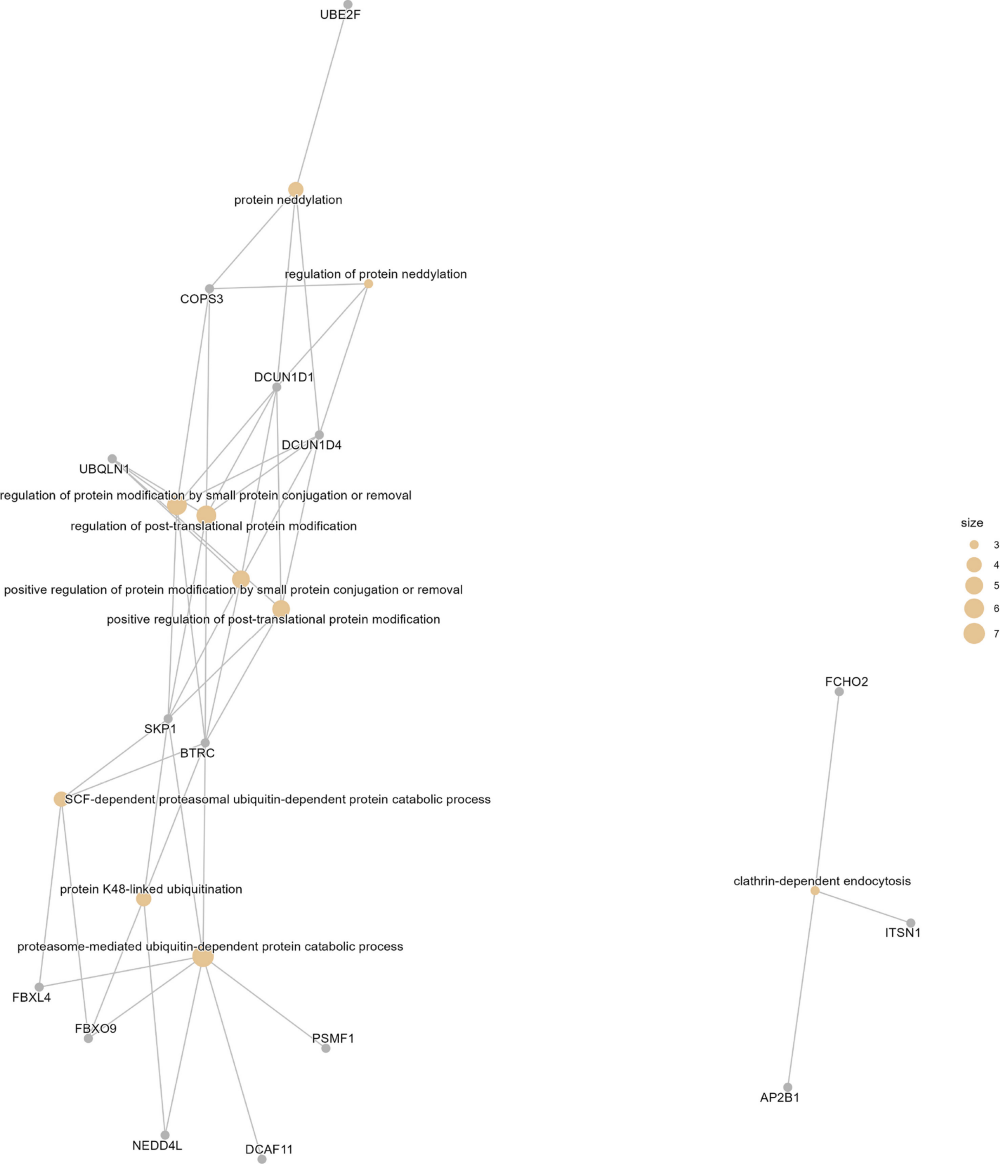
Gene network (cnetplot) of enriched Gene Ontology biological process (GO BP) terms from the 50 EPO‐responsive genes. Nodes represent GO terms (circles) or input genes (squares), with edges indicating gene term membership. The layout shows the relationships between the top enriched processes and their contributing genes.

Overall, the enrichment profile highlights a strong convergence on ubiquitin and neddylation‐dependent protein modification pathways, complemented by processes related to endocytosis, T cell signalling and RNA regulation (Figures 3 and 4).

#### 3.5.2 Exercise‐Responsive Genes

**FIGURE 5:**
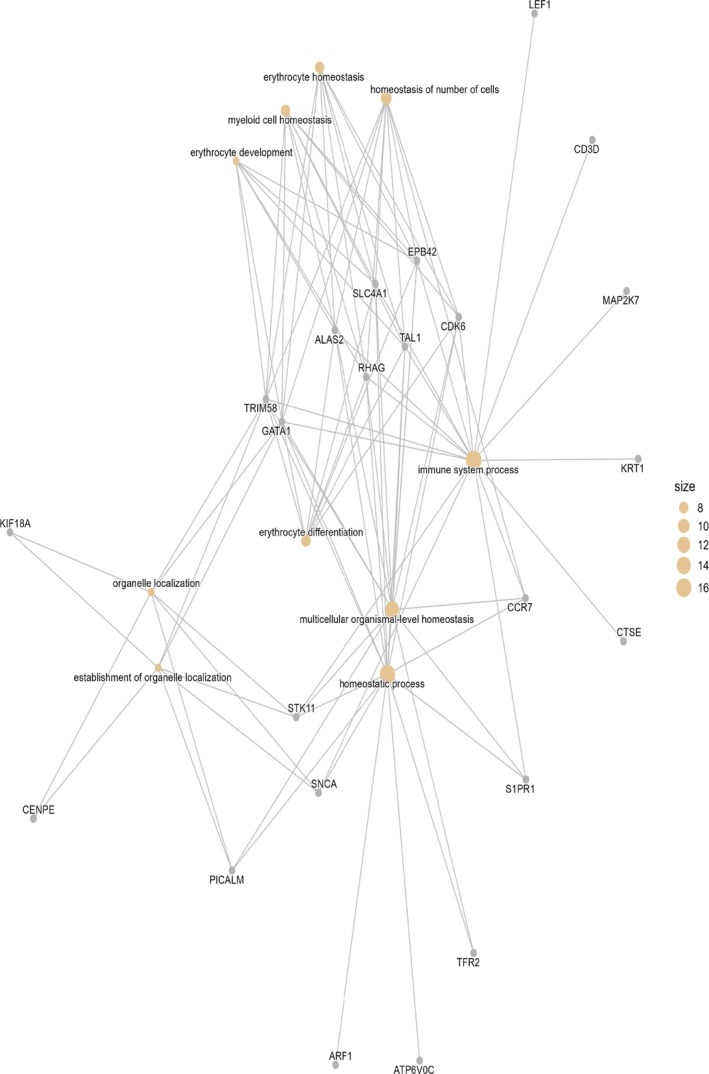
Network of significantly enriched biological process categories (*q* ≤ 0.05) identified from 34 exercise‐responsive genes, showing connections between genes (grey labels) and GO terms (orange nodes, sized by gene count).

**FIGURE 6:**
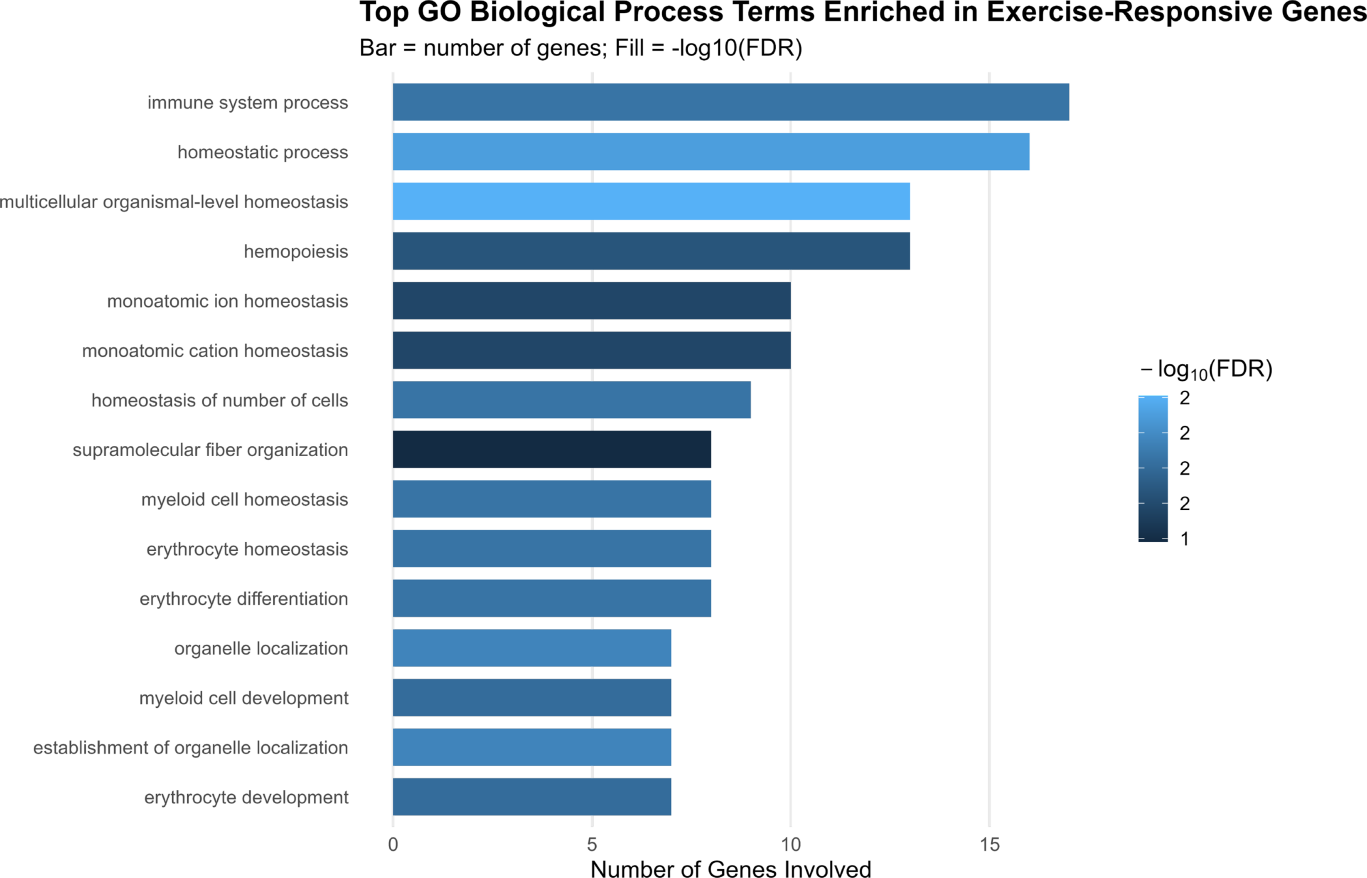
Top 13 GO biological processes enriched in exercise‐responsive genes. Bars show the number of genes per term; colour encodes −log_10_(FDR).

GO analysis of the 34 exercise‐responsive genes (tested against the measured background of 147 Entrez IDs) revealed significant enrichment for BPs related to homeostasis and haematopoiesis. The strongest associations were observed for multicellular organismal homeostasis (*q* = 0.0055), homeostatic process (*q* = 0.0071) and organelle localisation (*q* = 0.0100). Multiple terms linked to erythroid biology were significantly enriched, including erythrocyte differentiation, erythrocyte homeostasis and erythrocyte development (*q* ≤ 0.014). Additional processes included myeloid cell homeostasis and development, immune system processes (17 genes, *q* = 0.0126) and hemopoiesis (13 genes, *q* = 0.0196). Terms reflecting ion transport and regulation were also detected, such as monoatomic ion homeostasis and monoatomic cation homeostasis (*q* ≈ 0.024). Collectively, these findings indicate that exercise‐responsive transcripts are strongly enriched for pathways underpinning erythropoiesis, immune regulation and cellular homeostasis (Figures 5 and 6 and Table S9).

#### 3.5.3 Altitude‐Responsive Genes

**FIGURE 7:**
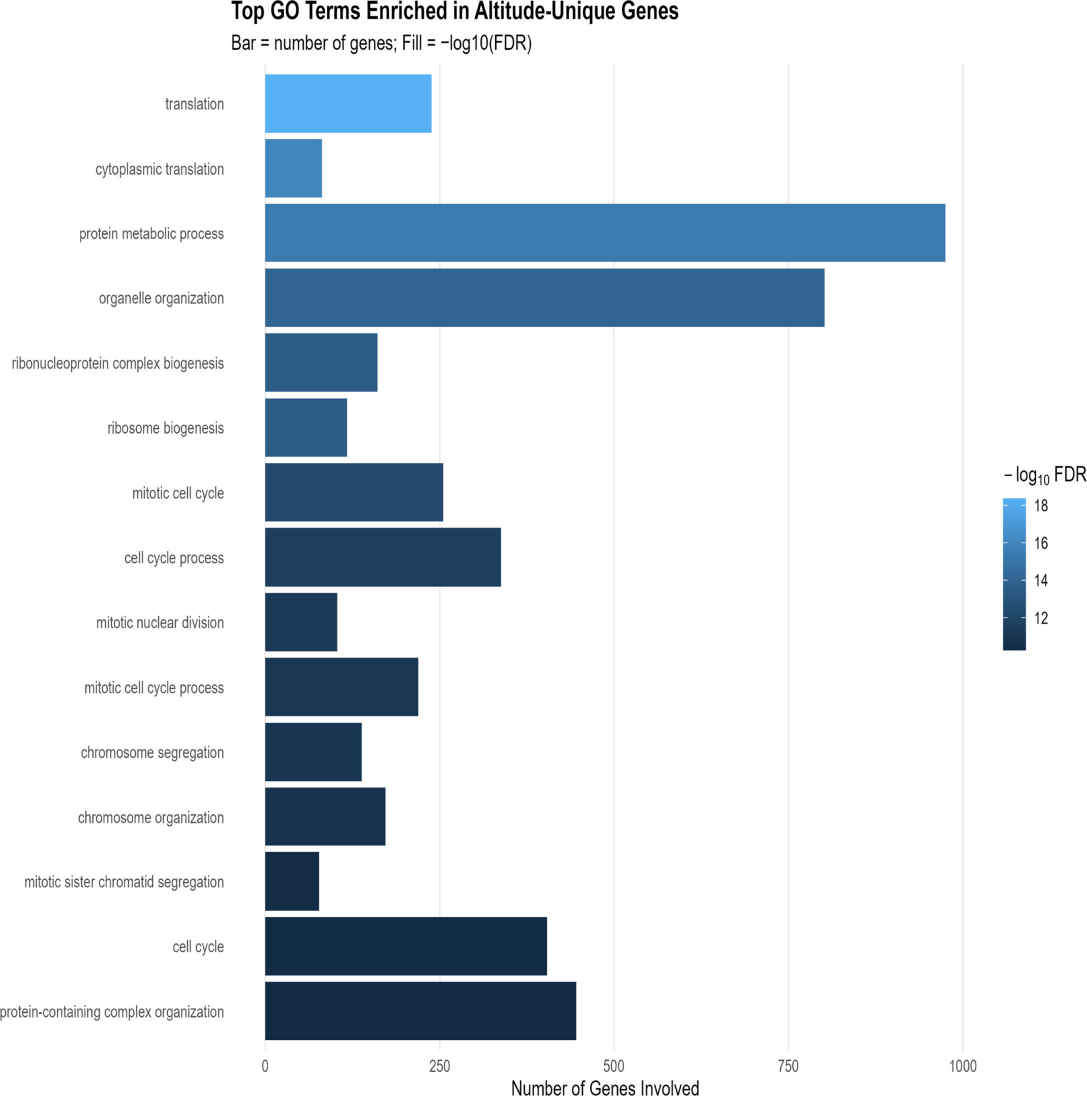
Top 15 GO biological processes enriched in Altitude‐responsive genes. Bars show the number of genes per term; colour encodes −log_10_(FDR).

**FIGURE 8:**
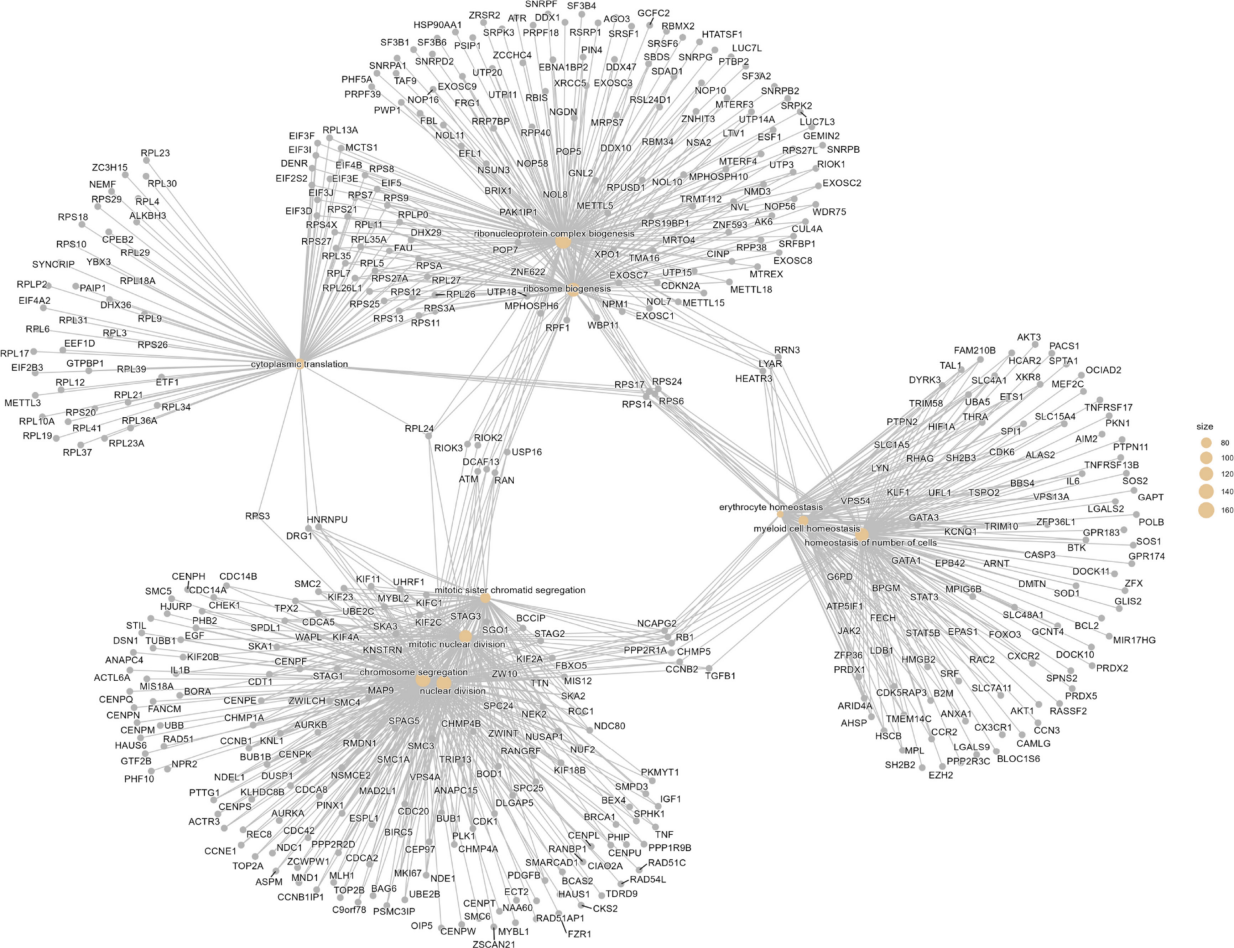
Altitude—Networks of the top 10 enriched biological processes and their associated genes, using a Kamada–Kawai layout.

Using the measured universe (3235 genes; background 18,744; BH FDR), the gene set was most strongly enriched for translation/RNA biogenesis, led by translation (GO:0006412; fold enrichment = 1.83; *z* = 10.66; FDR = 4.34 × 10^−19^; −log_10_FDR = 18.36) and cytoplasmic translation (2.78; *z* = 10.60; 1.14 × 10^−16^; 15.94), with concordant signals for ribonucleoprotein complex biogenesis (1.90; *z* = 9.20; 3.02 × 10^−14^; 13.52) and ribosome biogenesis (2.15; *z* = 9.38; 3.02 × 10^−14^; 13.52). Organelle/proteostasis programmes were also over‐represented, including organelle organisation (1.29; *z* = 8.82; 1.01 × 10^−14^; 13.99) and protein metabolic process (1.26; *z* = 9.16; 4.62 × 10^−16^; 15.33). A coordinated cell‐cycle/mitotic module was enriched, highlighted by mitotic sister chromatid segregation (2.31; *z* = 8.37; 4.46 × 10^−11^; 10.35), mitotic nuclear division (2.12; *z* = 8.63; 4.67 × 10^−12^; 11.33) and mitotic cell cycle (1.60; *z* = 8.59; 4.97 × 10^−13^; 12.30). Together, these results indicate a transcriptome biased towards translational machinery and ribosome assembly, alongside proteostasis and tightly coupled mitotic processes (Figures 7 and 8 and Table S9).

## 4 Discussion

We systematically assessed 153 candidate rHuEPO‐responsive genes across datasets representing pharmacological (rHuEPO), environmental (altitude) and physiological (exercise) erythropoietic stimulation. The objective was to define transcripts selectively modulated by rHuEPO, independent of natural EPO elevation through hypoxia or training. Failure to distinguish these responses is a recognised limitation in both the ABP and emerging transcriptomic approaches [9, 10]. By integrating standardised RNA‐seq profiles from the GEPREP database [19] with transcriptomic signatures from three independent altitude exposure studies [15, 16, 18], we looked at the extent of biological cross‐reactivity between rHuEPO administration [11, 13, 14] and common physiological stimuli. This approach provides a framework to refine candidate biomarker selection for future antidoping applications.

Environmental hypoxia (e.g., at altitude) triggers coordinated molecular and physiological responses that culminate in increased erythropoiesis [29]. Central to the stabilisation of hypoxia‐inducible factors (HIFs) is the inhibition of oxygen‐dependent prolyl‐hydroxylase domain (PHD) enzymes, which hydroxylate HIF‐α under normoxia but are inhibited when oxygen is limited [29]. In adults, HIF‐2α is the predominant regulator of EPO synthesis, acting in renal EPO‐producing cells (REPCs) and under moderate to severe hypoxia or pharmacological HIF activation in hepatocytes [29]. Transcriptional control is mediated by HIF binding to hypoxia response elements within the EPO regulatory regions [29].

HIF‐2α plays a pivotal role in iron metabolism, acting alongside other HIFs to coordinate erythropoiesis [30]. Hypoxia and HIF activity suppress hepcidin, while HIF‐regulated genes include transferrin (HIF‐1α), transferrin receptor‐1 (HIF‐1α) and ferroportin (HIF‐2α and hepcidin dependent) [30]. Together, these mechanisms increase iron availability for haemoglobin synthesis [30]. Such integration of oxygen sensing and iron regulation provides a plausible explanation for the similarity between transcriptional responses to altitude exposure and rHuEPO administration.

In this analysis, a substantial proportion of rHuEPO‐responsive genes were also regulated during altitude exposure. We observed 94 overlapping transcripts, many of which showed transient induction during the hypoxic period. Most changes returned to baseline after descent from altitude [15]. This suggests hypoxia‐induced expression is often reversible but still has the potential to overlap with gene expression profiles induced by rHuEPO administration. The finding is consistent with the central role of the HIF‐2–EPO–iron axis in both conditions. At high altitudes, hypoxia acts as the physiological trigger. In rHuEPO administration, the same axis is activated directly by exogenous hormone, independent of HIF‐2α [30].

EPO exerts its effects by binding to the EPO receptor (EPOR) on erythroid progenitors, activating the canonical JAK2/STAT5, RAS/ERK and PI3K signalling pathways [31]. Because both endogenous EPO and rHuEPO act through EPOR, these same cascades are expected to be engaged. In hypoxic or altitude conditions, the upregulation of endogenous EPO is primarily driven by the stabilisation of HIF‐2α [29, 30], whereas pharmacological rHuEPO dosing stimulates the receptor directly.

Altitude adaptation extends well beyond erythropoiesis. Storz [32] highlights that evolutionary and physiological studies demonstrate significant adaptations to high‐altitude environments. These include changes in ventilatory control, cardiovascular function, mitochondrial bioenergetics and immune regulation as key components of high‐altitude adaptation. These findings underscore that altitude‐induced adaptations engage a broad array of systemic, nonerythroid pathways. By contrast, rHuEPO is primarily associated with erythropoietic effects. From this perspective, the comparison between altitude adaptation and rHuEPO administration may help to differentiate systemic hypoxia‐driven responses from direct EPOR‐mediated signalling. The magnitude and duration of gene expression changes also differ. Altitude‐induced transcriptional responses usually normalise after descent [15]. In contrast, rHuEPO administration, including in microdosing regimens, produces sustained modulation of specific erythroid transcripts, with these changes persisting for weeks after plasma EPO levels have returned to baseline [11, 13]. Mechanistically, EPO signalling promotes proliferation and differentiation of erythroid progenitor cells [29, 31]. It provides a biological rationale for why transcriptomic changes can persist after dosing, as observed in rHuEPO administration studies [11, 13]. These temporal dynamics may have implications for antidoping efforts and justify further longitudinal validation.

In summary, the observed gene overlap between altitude exposure and rHuEPO administration is biologically plausible, as both rely on the HIF‐2–EPO–iron regulatory axis [29, 30]. At the same time, altitude adaptation involves a broader array of systemic responses [32] and differs in the temporal dynamics of gene expression compared with pharmacological EPO administration. These distinctions suggest opportunities to refine biomarker panels: for example, by deprioritising broadly hypoxia‐inducible transcripts and focusing instead on EPOR‐proximal or late erythroid genes that are less sensitive to environmental confounding.

The potential for exercise to influence the transcriptomic profile introduces an important consideration for the specificity of RNA‐based antidoping biomarkers. In a randomised, counterbalanced cross‐over trial, Dickinson et al. [33] profiled skeletal muscle after single bouts of AE and resistance exercise (RE) with biopsies at 1 and 4 h. By 1 h, both modes induced dozens of DEGs (AE = 48; RE = 67), and by 4 h, hundreds (AE = 221; RE = 523), highlighting substantial, mode‐dependent transcriptional activity. At 4 h, AE showed clusters involving zinc interaction, angiogenesis and ubiquitination. RE showed clusters involving transcriptional regulation, cytokine activity, cell adhesion and kinase activity. KEGG PI3K–Akt was uniquely enriched after RE. These findings underscore that exercise‐induced transcriptomic changes are non‐uniform and time dependent. The variability of exercise‐induced transcriptional responses was systematically quantified by Amar et al. [34]. A study showed a meta‐analysis of 43 studies and 59 cohorts, spanning both acute and long‐term interventions in skeletal muscle and blood. They detected 537 DEGs after acute exercise and 441 after long‐term training in skeletal muscle and 37 and 48 in blood, respectively. The analysis revealed that responses are moderated by age, sex and postexercise sampling time. SMAD3 emerged as a central regulator of the acute muscle response, validated in an independent cohort at 2 h post exercise [35]. These findings illustrate that transcriptomic signatures are shaped by multiple biological moderators, and while the authors did not study antidoping applications, such variability could complicate biomarker validation efforts.

In the context of the present study, these exercise datasets directly intersect with the 153 rHuEPO‐responsive candidate genes evaluated using GEPREP. For 34 of the 153 genes, our analysis revealed medium effect sizes (Cohen's *d* ≥ 0.5) in response to common exercise modalities, indicating a tangible risk of physiological cross‐reactivity. Pathways highlighted in Dickinson et al. [33] and Amar et al. [34] show considerable overlap with processes central to energy metabolism, angiogenesis and immune regulation domains, also targeted by rHuEPO administration. Although erythropoiesis is not explicitly reported, related signalling networks are involved. Without systematic filtering against high‐quality exercise datasets, there is a clear risk that physiological training adaptations could be misclassified as doping signatures. These observations reinforce the need for stronger specificity testing of transcriptomic biomarkers under varied physiological conditions.

Exercise, whether acute or chronic, aerobic or resistance, is a consistent and potent driver of gene expression changes in both skeletal muscle and blood. By integrating large‐scale resources such as GEPREP with targeted biomarker discovery pipelines, it becomes possible to identify and exclude transcripts that respond to training stimuli. Doing so is essential to minimise Such temporal differences could be valuable discriminators for antidoping purposes, erythropoietic stimulation.

The 50 EPO unique responsive genes identified in this study were significantly enriched in BPs. They are related to post‐translational protein modification, particularly within the ubiquitin–proteasome system (UPS), and in vesicle‐mediated trafficking pathways such as clathrin‐dependent endocytosis. The lack of overlap between these 50 EPO unique genes and those altered by altitude exposure or exercise in our dataset is notable. It suggests that their transcriptional regulation is less susceptible to common physiological stimuli known to confound antidoping biomarkers. This specificity increases their potential value in distinguishing EPO administration from environmental or training‐related adaptations. Key UPS components included E2 ubiquitin conjugating enzymes (e.g., UBE2F), Cullin–RING ligase adaptors (DCUN1D1, DCUN1D4, FBXO9, FBXL4 and BTRC) and proteasome regulators (PSMF1, COPS3 and UBQLN1).

During human terminal erythroid differentiation, the deubiquitylase USP7 is upregulated [35]. Genetic knockdown or pharmacological inhibition of USP7 impairs late‐stage maturation. Reduced glycophorin A (GPA) expression, delayed loss of α4‐integrin, decreased Band 3 and γ‐globin expression, defective enucleation and increased apoptosis [35]. Mechanistically, USP7 directly interacts with the erythroid transcription factor GATA1, removes K48‐linked polyubiquitin chains and stabilises GATA1 by preventing its proteasomal degradation [36]. Catalytically inactive USP7 does not stabilise GATA1, and the loss of GATA1 following USP7 inhibition is rescued by the proteasome inhibitor MG132 [35]. In addition, KLF1 levels decrease after USP7 knockdown, consistent with its regulation by GATA1. Collectively, these findings demonstrate that USP7 regulates terminal erythroid maturation by maintaining GATA1 stability [35].

EPO–EPOR signalling activates the JAK2–STAT5 pathway, which integrates with master regulators such as GATA1 and KLF1 to drive erythroid gene programmes. Genome‐wide enhancer profiling showed that EPO stimulation rapidly remodels the enhancer landscape, altering histone acetylation at thousands of regions. These enhancers are enriched for GATA1, KLF1, TAL1/E‐box and STAT5 motifs and include super‐enhancers linked to key erythroid regulators [36, 37]. This enhancer architecture highlights how EPO signalling coordinates chromatin dynamics with transcriptional control during erythropoiesis.

The identification of clathrin‐associated factors (AP2B1, ITSN1 and FCHO2) highlights regulated endocytosis as an additional pathway linked to EPO response. AP2B1 encodes the β2 subunit of the AP‐2 clathrin adaptor complex, which recognises cargo sorting signals and connects them to the clathrin coat during vesicle formation [38]. FCHO2 is required for the nucleation of clathrin‐coated pits and marks their initiation sites [39]. ITSN1 functions as a multidomain scaffold that contributes to organising endocytic protein networks, including AP‐2, and is stably enriched at perisynaptic endocytic zones where it supports early vesicle formation events [40].

EPOR internalisation via clathrin‐coated vesicles is a key mechanism for regulating the duration of receptor signalling [41]. Ligand‐induced EPOR endocytosis requires JAK2 kinase activity and specific cytoplasmic tyrosines (Y429, Y431 and Y479), which recruit the p85 subunit of PI3K, acting in a PI3K activity–independent manner to link EPOR to the endocytic machinery [41]. Bulut et al. [42] further showed that Cbl‐mediated ubiquitination of p85 enables its interaction with the clathrin adaptor epsin‐1, driving efficient receptor endocytosis. Defects in this pathway are evident in primary familial and congenital polycythaemia (PFCP). Sulahian et al. [41] showed that reduced EpoRs from PFCP patients, which lack key cytoplasmic tyrosine, fail to bind p85 and do not internalise upon stimulation, contributing to EPO hypersensitivity and prolonged signalling. Bulut et al. [42] further demonstrated that PFCP‐mimicking EpoRs cannot activate the Cbl/p85/epsin‐1 pathway, fail to colocalise with epsin‐1 and also exhibit EPO hypersensitivity in primary erythroid progenitors.

Liang et al. [35] showed that the deubiquitylase USP7 regulates terminal erythroid maturation by stabilising the transcription factor GATA1. Loss of USP7 function, either by genetic knockdown or pharmacological inhibition, impaired late‐stage erythroid. Differentiation, underscoring the importance of UPS regulation in red blood cell development.

The lack of overlap between many of these EPO‐responsive genes and those altered by exercise or hypoxic altitude exposure in our dataset reinforces their potential value as relatively specific biomarkers. Exercise‐induced gene expression changes often unite on metabolic adaptation, oxidative stress responses, and cytoskeletal remodelling. Meanwhile, altitude exposure strongly induces HIF target genes related to angiogenesis, glycolysis and oxygen transport. In contrast, the 50 gene EPO subset captured here is dominated by regulatory machinery for targeted protein degradation and intracellular trafficking, which may not be activated to the same extent by physiological stimuli.

Recent work by Loria et al. [43] provides complementary validation of the biomarker potential of ALAS2 and CA1. In their longitudinal study, ALAS2 and CA1 expression increased significantly following rHuEPO microdosing, by ~300% and ~200%, respectively. Sensitivity reached 97%–98% and specificity 95%–100% at both sea level and altitude. These findings highlight the considerable promise of RNA‐based candidate biomarkers. In our analysis, ALAS2 and CA1 are reported as responsive biomarkers to rHuEPO but also in studies of altitude and exercise. Loria et al. [43] confirmed their strong responsiveness under controlled rHuEPO administration. However, their responsiveness to physiological stressors underscores the need for careful validation to confirm that elevations reflect rHuEPO misuse rather than natural adaptation. Therefore, ALAS2 and CA1 illustrate both the promise and the challenges of biomarker validation. In comparison, our 50 EPO‐responsive candidate genes showed no overlap with altitude or exercise datasets, suggesting resistance to these common stressors. Future work should evaluate both categories: EPO‐specific candidates that appear robust against confounding and overlapping genes such as ALAS2 and CA1 that show strong responsiveness and large effect sizes. A combined approach, validated across diverse cohorts and conditions, will be essential to establish a reliable biomarker panel for real‐world antidoping frameworks.

## 5 Limitations

This study has several limitations. Analyses using the GEPREP database were restricted by its “black box” design. Although outputs are uniformly processed, users cannot access raw counts or key metadata such as study identifiers, sample sizes or time points. The altitude datasets were also heterogeneous, employing different sequencing platforms, exposure durations, statistical thresholds and participant characteristics, which limits direct comparability. In addition, certain exercise groups within GEPREP had small sample sizes, reducing statistical power and increasing the risk of false negatives. While some datasets were aligned to the hg19/GRCh37 reference genome, we harmonised all gene symbols against the current HGNC‐approved nomenclature GRCh38 (including historical aliases and updated entries). This ensured that no genes were lost due to annotation drift. Taken together, these limitations underscore the need for harmonised raw datasets and standardised analytical pipelines to enable stronger cross‐study comparisons in future biomarker discovery.

We also chose not to present unified fold‐change rankings or cross‐study heatmaps. The rHuEPO, exercise and altitude datasets were generated on different platforms (RNA‐seq vs. microarray), at non‐aligned time points and with inconsistent statistical frameworks. Moreover, the GEPREP exercise database does not report fold‐change values, making it impossible to assess effect magnitude and further restricting comparability. For these reasons, we restricted our integration to gene overlaps and pathway‐level analyses, which are more robust to such methodological differences. Transcriptomic profiling represents the first realistic opportunity to extend the ABP beyond haematology since its introduction in 2009. With our group now piloting RNA extraction from DBS, this pathway may become technically achievable soon.

## 6 Conclusion

This study identified 50 gene transcripts that are responsive to rHuEPO but remain stable under both exercise and altitude exposure. These biomarkers demonstrated strong specificity, minimal physiological confounding and enriched pathways unrelated to hypoxia‐induced erythropoiesis. By systematically excluding genes affected by common training or environmental conditions, we present a refined panel of promising candidate transcriptomic markers with potential for use in antidoping applications. These findings represent an important step towards improving RNA‐based detection methods, but experimental validation is still required to confirm their robustness and applicability in real‐world settings.

## Author Contributions

*Conceptualisation*: D.O. and Y.P. *Formal analysis*: D.O. and Y.P. *Methodology*: D.O. and Y.P. *GEPREP results*: D.O. and L.L. *Writing – original draft*: D.O. and Y.P. *Writing – review and editing*: D.O., S.S., Z.Z and Y.P.

## Conflicts of Interest

The authors declare no conflicts of interest.

## Supporting information


**Table S1:**. Gene symbol harmonisation, dataset source, overlap with GEPREP and altitude datasets and expression direction of rHuEPO‐candidate genes. Public datasets in GEPREP were aligned to GRCh37/hg19 (2013) and earlier rHuEPO studies (e.g., Durussel et al. [9] and Wang et al. [11]) used historical gene nomenclature; gene symbols were harmonised to current HGNC symbols (HGNC December 2024; Ensembl v111 and NCBI Gene accessed 2025), and only genes with an official symbol change are listed in the harmonisation section. The subsequent section lists 153 rHuEPO‐candidate genes with their source rHuEPO dataset/study and indicates whether each gene overlaps with GEPREP and/or altitude datasets (including the relevant altitude study where overlapping). A separate section summarises expression direction for rHuEPO and altitude datasets (up/down/mixed, with time points where available); direction is not reported for GEPREP because fold change/log_2_ fold change is not provided in GEPREP.

**Table S2:** Altitude gene overlap and study‐level presence across Manella, Pham_HA1, Pham_HA3 and Sutehall. Pairwise overlap count matrix and gene‐level presence table for altitude‐responsive genes across Manella, Pham Day 1 (Pham_HA1), Pham Day 3 (Pham_HA3) and Sutehall. ^†^Pairwise overlap count matrix summarises shared altitude‐responsive genes across studies; diagonal values indicate the total number of genes reported per study, and off‐diagonal values indicate the number of overlapping genes between each pair of studies. ^‡^The gene‐level presence table lists harmonised HGNC symbols and study‐level presence (TRUE/FALSE) for each altitude dataset; n_altitude_studies indicates the number of altitude studies in which a gene is present, and genes_in_more_than_one_altitude_study is TRUE when a gene is present in ≥ 2 altitude studies.

**Table S3:** GEPREP screening of 153 rHuEPO‐candidate genes for exercise significance, with Cohen's *d* calculated for *p* < 0.05 and/or NaN‐returning genes. GEPREP‐derived exercise outputs for all 153 rHuEPO‐candidate genes of interest (i.e., the full rHuEPO gene list assessed in GEPREP) to determine whether each gene shows evidence of exercise‐related significance. Cohen's *d* is reported for genes meeting the predefined calculation criteria (*p* < 0.05 and/or genes returning NaN values) to support evaluation of biological relevance.

**Table S4:** Sutehall et al. high‐altitude transcriptomic dataset (29 significant DEGs). Sutehall normalised data provide the normalised expression values for the 29 significant DEGs across the reported time points/conditions in the Sutehall dataset, presented to enable transparent inspection of the magnitude of gene expression changes within this altitude study. Cohen's *d* reports effect sizes calculated for the same 29 significant DEGs to assess biological relevance, applying *d* ≥ 0.5 as the predefined threshold for a moderate effect.

**Table S5:** Pham et al. altitude differential expression outputs (Days 1 and 3). Altitude‐responsive differential gene expression results from Pham et al., reported separately for samples collected on Days 1 and 3 of altitude exposure. Results are shown in two blocks (Days 1 and 3), with the same output fields reported for each day and gene identifiers included to support harmonisation and downstream overlap analyses.

**Table S6:** Manella dataset output and summary. The table is presented in five sections: RI vs. SL, PU vs. SL and RI vs. PU (each reporting Gene, log2FoldChange, lfcSE and stat); SL, PU and RI (reporting Gene, baseMean, pvalue and padj); and a summary/filter section reporting Gene, log2FC, Pass and the altitude interval label (200–3800M, 200–5100M or 3800–5100M) with PASS/FAIL text shown in the table.

**Table S7:** Sharma and Sethy reported altitude‐related gene list (gene symbols only; no DEG results available).

**Table S8:** Summary counts describing the overlap structure between rHuEPO‐responsive candidate genes (EPO), the altitude‐responsive gene panel (ALT) compiled from reproducible hypoxia studies and exercise‐responsive candidate genes (EXE) identified from the GEPREP evaluation. Counts are shown for unique sets and intersections to document how genes were categorised prior to downstream analyses and Gene Ontology (GO) enrichment.

**Table S9:** GO enrichment analysis of rHuEPO, altitude and exercise‐responsive genes. GO biological process enrichment was performed separately for each gene set: rHuEPO‐responsive candidate genes (EPO), altitude‐responsive genes compiled from reproducible hypoxia studies (ALT) and exercise‐responsive candidate genes identified from the GEPREP evaluation (EXE). The table reports GO term ID and Description, GeneRatio (input genes annotated to the term/total input genes), BgRatio (background genes annotated to the term/total background genes), RichFactor (GeneRatio/BgRatio), FoldEnrichment (over‐representation relative to background), Enrichment score (if reported by the analysis), *p* value, p.adjust (multiple‐testing adjusted *p* value), qvalue (false discovery rate, FDR), geneID (contributing genes) and Count (number of input genes mapped to the term).

## Data Availability

The data that support the findings of this study are available on request from the corresponding author. The data are not publicly available due to privacy or ethical restrictions.
